# Thank you to all of our reviewers in 2021

**DOI:** 10.1038/s42003-022-03145-x

**Published:** 2022-02-28

## Abstract

5514 individual reviewers contributed to the peer review process at *Communications Biology* in 2021. We acknowledge and thank each of them here.

Many of us thought—or at least hoped—that the COVID-19 pandemic would be far behind us by 2022. Unfortunately, as we all know, this hasn’t been the case. We continue to live with a huge amount of uncertainty and unpredictable disturbances to our normal schedules. Yet, in the face of all the challenges imposed by the pandemic, science marches on. Just over 3100 manuscripts were submitted to *Communications Biology* in 2021 and 58% of those were sent for external review. In total, we received 7877 review reports, including both new submissions and revised manuscripts. We are extremely grateful to the more than 5000 individual reviewers who volunteered their time and expertise to help evaluate these manuscripts and thus contributed to the advancement of science beyond their own personal research programs.

Yet, in the face of all the challenges imposed by the pandemic, science marches on.

## Reviewers of the Month

Each month our editors select one of the many excellent reviewers we have worked with at any point in the past to be featured as Reviewer of the Month. Reviewers are selected who have provided exceptional advice to authors and/or editors and who the editors feel went above and beyond in their peer review duty. To learn more about our Reviewers of the Month, please see our website.

**January:** Nicholas Marra, Drury University, USA

**February:** Kaoru Ito, RIKEN Center for Integrative Medical Sciences, Japan

**March:** Ilhem Messaoudi, University of California Irvine, USA

**April:** Umberto León-Domínguez, University of Monterrey, Mexico

**May:** Dipanjan Roy, National Brain Research Center, India

**June:** Hisashi Kawasaki, University of Tokyo, Japan

**July:** Anna Martner, University of Gothenburg, Sweden

**August:** Katrina Jones, University of Manchester, UK

**September:** Alex Moffett, York University, Canada

**October:** Brian Steidinger, University of Konstanz, Germany

**November:** Shelly Buffington, The University of Texas Medical Branch, USA

**December:** Tinatin Brelidze, Georgetown University Medical Center, Washington, D.C., USA

## All 2021 Reviewers

All individuals who submitted a report in 2021 are recognized below in alphabetical order by surname. We remain truly grateful to all of our reviewers. As always, journal staff take full responsibility for any misspellings, omissions, or other errors.

Reviewers who submitted 4 or more reviews in 2021 are highlighted in bold font and the number of reviews submitted is shown in brackets.

Duur K. Aanen, Adam Abate, Mostafa Abdelrahman, Maha Abdullah, Masato Abe, Hossam Abelsamed, Giancarlo Abis, Sherif Abou Elela, Wassim Abou-Kheir, Gad Abraham, Andrey Abramov, Dina B. AbuSamra, Adele Adamo, David J. Adams, Andrew C. Adey, Abhijin Adiga, James Adjaye, Meshack Afitlhile, Behrouz Aflatoonian, Behdad Afzali, Saurabh Agarwal, Michail Agathokleous, Chinyere Agbaegbu Iweka, Sara Aghajari, Miriam Agler-Rosenbaum, Melina A. Agosto, Irina Agoulnik, Alok Agrawal, Devendra K. Agrawal, Cristina Aguayo-Mazzucato, Aitor Aguirre, Adriano Aguzzi, Golam Ahammed, Ivan Ahel, Per Ahlberg, Hajar Fauzan Ahmad, Rafi Ahmad, Mohammad R. Ahmadian, Yasir Ahmed-Braimah, Atique Ahmed, Sheraz Ahmed, Wylie Ahmed, Jeonghyun Ahn, Johanne Ahrenfeldt, Gaurav Ahuja, Shintaro Aibara, Catherine Aime, Katherine Aird, Takashi Akera, Klaus Aktories, David Alabadi, Amal Alachkar, Alejandro Alagón, Gregorio Alanis-Lobato, Gregory F. Albery, Mattia Albiero, Urs Albrecht, Matteo Aldeghi, Lauren Aleksunes, Riccardo Alessandro, Matthew Alexander, William H. Alexander, Andrei Alexandrescu, Mohamed Alfaleh, Lital Alfonta, Azahar Ali, Matthew Aliota, Celeste Allaband, Micah Allen, Philip Allen, William Allen, Pierre Aller, Christoph Allolio, Luke Allsopp, Catarina R. Almeida, Mads Almose Røpke, Noelia Alonso Gonzalez, Roberto Alonso-Matilla, John Alroy, Mohamed Alsharedi, Abdulraheem AlShareef, Andrew Alspaugh, David Alsteens, Myriam Altamirano-Bustamante, Eckart Altenmüller, Claudia AlteriI, John Altin, Sandra Álvarez-Carretero, Alicia Alvarez, Mariano Alvarez, Jose Carlos Alves-Filho, Yahyah Aman, Rama R. Amara, Ivano Amelio, Sarah R. Amend, Claudio Ametrano, Shady A. Amin, Elissa M. Aminoff, Ariel Amir, Christoph Anacker

José Anadón, Ranjan Anantharaman, Frank Anderson, Kenneth C. Anderson, Nathan D. Anderson, Richard Anderson, Alma Andersson, Sarah Andres, Frank Angenstein, Hernan Anllo, David K. Ann, Migliaccio Anna Rita, Jitka Annen, Jean Sebastien Annicotte, Mark Anthony, Neal Anthwal, **Srdjan D. Antic [4]**, Michael Antle, Kazuhiro Aoki, Scott T. Aoki, Lina Aoyama, Luiza Aparecido, Samuel Aparicio, Hannah Apostolou, Kazuharu Arakawa, Beatrice Aramini, Yennyfer Arancibia, Mehdi Arbabi-Ghahroudi, Joseph F. Arboleda-Velasquez, Mariona Arbonés, Fabrice Arcizet, Alvaro Ardiles, Catalina Arévalo Ferro, David Argyle, Ehtesham Arif, Takahiro Arima, Shin-ichi Arimura, Ivan D. Arismendi, Jean-Paul Armache, Andrea Armani, Elizabeth Arnold, Leggy Arnold, Serge Aron, Joaquín Arribas, Roberto Arrighi, Janelle C. Arthur, Prabhu Arunachalam, Hiroshi Asahara, Munehiro Asally, Dominik Aschenbrenner, Kaveh Ashrafi, Ana Paula Assis, Richard Assoian, Mariana Astiz, Peter Atkinson, Ilaria Attili, Mukundan Attur, Scott X. Atwood, Edmund Au, Kin Au, Shannon Wing Ngor Au, Jane Aubin, Valentina Audrito, Erik Aurell, Didier Aurelle, Noam Auslander, Kellar Autumn, José L. Avalos, Bruno B. Averbeck, Karen Avraham, Ola Awad, Yimon Aye, Frank Aylward, Ugur Ayturk, Jose M. Ayuso, Tomas Babak, Jodie L. Babitt, Gopal Babu, Dominik Bach, Robert Bachoo, Steffen Backert, Myriam Baes, Nicolas Baeyens, Ulas Bagci, Nader Bagheri, Yoann Bagousse-Pinguet, Peter Bai, Xiaochen Bai, Mirza (M.F.A) Baig, Joseph D. Bailey, Travis Bailey, George Baillie, Arnold Bainomugisa, Denis Baird, Claude J. Bajada, Prabin Bajgain, Adam Bajgar, Monika Bajorek, Anna Bakenecker, Bradley J. Baker

Matthew A. Baker, Jeroen Bakkers, Diana Balazsfi, Gábor Balázsi, Diego Balboa, Cosima T. Baldari, Daniel Baldauf, Nicholas L. Balderston, Megan Baldridge, Philip Baldwin, Stefan Balint, Pedro Ballester, Detlef Balschun, David A. Baltrus, Tiziano Balzano, Rakesh Bam, Laura Banaszynski, Gavin Band, Kanan Bando, Jean-Louis Banères, Manidipa Banerjee, Ronadip Banerjee, Botond Banfi, Duhee Bang, Marie-Louise Bang, Samuel Banister, Vikas Bansal, Shilai Bao, Yongping Bao, György Barabas, Joan Barau, Nicola Barban, Sandrine Barbaux, Jayme Barbedo, Annika Barber, David A. Barbie, Aurel Barbotin, Olga Barca, Mary Helen Barcellos-Hoff, Mira Barda-Saad, Marin Barisic, Alice Barkan, Richard Barker, Michalis Barkoulas, Vincenzo Barnaba, Samuel Barnes, Byron Baron, Celia Baroux, Rodrigo Barquera, Rodolphe Barrangou, Christopher Barratt, Ellen F. Barrett, Jeffrey Barrett, Arantza Barrios, Jaime Barros-Rios, Jordan Barrows, Daniel J. Barshis, Gregory Barshtein, Alexander Bartelt, Marine Barthez, **Francesca Bartolini [4]**, Carl Barton, Nir Barzilai, Sujit Basak, Chris Bashur, Franco Basile, Jean-Etienne Bassard, Jonelle Basso, Julien Bastin, Joyoti Basu, Wagner Batista, Alexandra Battaglia-Mayer, Andrea Battisti, Davide Battisti, Fabia Battistuzzi, Lee Baugh, Anthony D. Baughn, Sophie Bavard, Sina Bavari, Vassiliy N. Bavro, Pushpinder Bawa, Omer Faruk Bayrak, Fuller Bazer, Stuart Bearhop, Rachel Bearon, Megan Beaudry, Audrey Beaussart, Melissa Beck, Catherina G. Becker, Christine Becker, Donald Becker, Hendrik Beckert, Justin Bedo, Díaz Begoña, Samuel M. Behar, Mira Behnam, Hamid Behravan, Hassannia Behrouz, Hamid Beiki, Eric Beitz

Nadjet Belbachir, Benjamin Belgrad, Christopher Bell, Michele Bellingeri, Jacob Bellmund, Nicholas Bello, Camilla Bellone, Georgiy A. Belogurov, Alexandre Belot, Jennifer E. Below, Aniruddha Belsare, Gabrielle Belz, Yaacov Ben-David, Gustavo Benaim, Barbara Benassi, Carla F. Bender Kim, Andrew Bender, Elisabetta Benedetti, Thomas Beneyton, Valentina Benfenati, Kingsly Beng, Johan Bengtsson-Palme, Itai Benhar, Yoselin Benitez-Alfonso, Andreana Benitez, Blas Benito, Collyer Benjamin, Jada Benn Torres, Joe Bennett, Isabelle Benoit Gelber, Roland G. Benoit, Olivier Bensaude, J Kelley Bentley, Ivan Berg, Tobias Berg, David Berger, Philipp Berger, Thomas K Berger, Christopher Bergevin, Albert M. Berghuis, Helmut Bergler, Andreas Bergthaler, David B. Berkowitz, Ben Berks, Ilana Berlin, Antonio Bernad, Antonietta Bernardo, Dirk Bernhardt-Walther, Anne Bernheim-Groswasser, Aude Bernheim, Jimena Berni, Annie Bernier, Melissa M. Berrien-Elliott, Matthew Berriman, Carole Berruyer, Luiz Eduardo Bertassoni, Thomas Bertero, Stefano Berto, Camilla Bertolini, Giorgio Bertorelle, George Bertsias, **Luciana Besedovsky [4]**, Robert Best, Christian Beste, Dany Beste, Sophie Bestley, Lia Betti, Viviana Betti, Richard F. Betzel, Gabriel Bever, Anna Beyeler, Evgeny E. Bezsonov, Aparna Bhaduri, Kapil Bharti, Aadra Bhatt, Arjun Bhattacharya, Martha R. Bhattacharya, Samarjit Bhattacharyya, Sudeepa Bhattacharyya, David Bhella, Pradeep Bhide, Yoshita C. Bhide, Yanzhen Bi, Tommaso Biancalani, Enrica Bianchi, Tina Bianco-Miotto, Lucia Biasutto, Ulf Bickmeyer, Martin Bidartondo, Ralf Biehl, Anna Bielak-Zmijewska, Monika Bielecka, Jana Biermann, Kasia Bieszczad, Paola Binda, Mario Binelli, Alexander Birbrair, James J. Birchler, Rasmus M. Birn, James Birrell

Anthony Bishop, Melanie Bishop, Kateřina Bišová, Nea Bister, Bharat Biswal, Bichitra K. Biswal, Haimanti Biswas, Kyle Bittinger, Lise Bjørkhaug, Crysten Blaby-Haas, Jennifer Black, Joshua Black, Harvey Blackburn, Jonathan M. Blackburn, Seth Blackshaw, Julie Blackwood, Sarah Blagden, Stephen Blake, Stéphane Blanc, Charles Blanch-mercader, Francisca Blanco Herrera, Philippe Blancou, Wulf Blankenfeldt, Robert E. Blankenship, Sonja Blasche, Mark Blaskovich, Miguel A. Blazquez, Camille Bleriot, Dieter Blottner, Cedric Blouin, Lars Bock, Ralph Bock, Rich Boden, Till Boecking, David Boehr, Ralph T. Boettcher, Erik C. Boettger, Alexander Bogachev, Rafal Bogacz, Matthew Bogyo, Jen Bohon, Michele Boiani, Brigitte Boizet-Bonhoure, Elvan Boke, Lukasz Bola, Andreas Boland, Maura Boldrini, Andrea Bolognesi, Guillaume Bompard, Will Bone, Charlotte M. Bonefeld, Martin Bonin, Nancy M. Bonini, Amélie Bonnefond, Susan Bonner-Weir, Morgane Boone, Panadda Boonserm, David Booth, Thomas Boothby, Alisdair B. Boraston, Enrica Bordignon, Bart Borghuis, Larry Borish, Maria Borisova-Mubarakshina, Carola Borries, Piet P. Borst, David A. Borton, Antoni J. Borysik, Oliver Bosch, Somdeb BoseDasgupta, Francesca Bosisio, Kristopher Bosse, Mihnea Bostina, Lucas Eduardo Botelho de Souza, João Botelho, Eva Böttcher- Friebertshäuser, Christina Boucher, Julie Bouckaert, Paul Bourdeau, Mathieu Bourdenx, Thomas Bourguignon, Jason Bourke, Travis Bourret, Sophie Bouton, Samuele Bovo, Robert Bowser, Mary Boyce, Claire Braboszcz, Ryan Bracewell, Andreas Bracher, Heather Bracken-Grissom, William J. Brackenbury, Laura Bradfield, Todd Bradley, Susan D Brain, Katherine Brakora, Janita Bralten, Mattia Bramini, Guo-Jie Brandon-Mong, Hans Brandstetter

Maarten M. Brandt, Christopher Braun, Martin Brazeau, William Brazelton, Laurent Bréhélin, Tinatin I. Brelidze, Fion Bremner, Cormack P. Brendan, Volker Brendel, Faith H. Brennan, Fabiana Bressan, Stéphane Bressanelli, Francois Bretagnolle, Sophie Breton, Damien Brevers, Hannah Bridges, Dora Brites, Luiz Brito, Vania Broccoli, Christopher A. Brochu, Chad Brock, Susan Brockerhoff, Emelie Brodrick, Paul S. Brookes, Jeffrey Brooks, Jan J. Brosens, Peter Bross, Grant Broussard, Isabelle Broutin, Andrea Brovelli, Anne M. Brown, Anthony Brown, Christopher J. Brown, Michael F Brown, Nathaniel Brown, Teagan Brown, Shane Browne, Douglas F. Browning, James Brozik, Patricia L. Brubaker, Melanie Bruchard, Jason Bruck, Joseph Bruckner, Marjorie Bruder, Thomas Brueser, Joan S. Brugge, Pierre Bruhns, Teodor-D. Brumeanu, Tilman Brummer, Stephen L. Brusatte, Camron Bryant, Donald A. Bryant, Ola Brynildsrud, Robert J. Bryson-Richardson, Nicole Buan, Mariarosaria Bucci, Susan K. Buchanan, Matthias Buck, Ann Bucklin, Hanna Budayeva, Cliff Bueno de Mesquita, Rochelle Buffenstein, Shelley Buffington, Katrine Bugge, Stéphane Buhler, Philippe Bulet, Michael Bulger, Alex N. Bullock, Christopher Bunick, Marianne Burbage, Christoph Burckhardt, Kathryn P. Burdon, Kyle Burger, Elizabeth Burgess, Liana Burghardt, Katja Burk, Kristopher Burkewitz, Emily Burnside, Christopher Burridge, Anne Burrows, Judith Burstin, David W. Burt, Maxwell Burton-Chellew, Adam Burton, Marc Aurel Busche, Sergei Butenko, Evan Byrnes, James R. Byrnes, Adam Byron, Dominika Bystranowska, Cesar Caballero Gaudes, Joana Cabral, Delia Cabrera DeBuc, Jilda A. Caccavo, Martin Caffrey, Danfeng Cai, Ling Cai, Shijie Cai, Yongfei Cai, Laia Caja

Antonio N. Calabrese, Mauro Calabrese, Luz Irina Calderón Villalobos, Heather Caldwell, Sara Calhoun, Erin S. Calipari, Damien Callahan, Fabiano Calmasini, Beatriz Calvo-Merino, Antonella Camaioni, Jodi Camberg, Alexander D. Cameron, Carlo Camilloni, Emma Camp, Leonardo Campagna, Hamish Campbell, Chiara E Campiglio, Teresa Campillo Ferrer, Javier Canales, Julián Candia, Angela Cánovas, Brandi L Cantarel, Margherita Cantorna, Claudio Cantù, Cara Cao, Hongnan Cao, Jicong Cao, Liu Cao, Xiang Cao, Melania Capasso, Daiana Capdevila, Daiana A. Capdevila, Natasha Caplen, Noel Caplice, Rob Capon, Vito Carlo Caponio, Andrea Caporali, Ornella Cappellari, Giovanni Cappello, Gerarda Cappuccio, Emidio Capriotti, Giulio Caravagna, Pablo Carbonell, Susanna Cardell, Cesar Cardenas, Andrea Cardini, **Marcelo Cardoso dos Reis Melo [10]**, Marica Cariello, Alexandre F. Carisey, Jeremy Carlier, Tomás Carlo, Luís D. Carlos, Maryn Carlson, Jeremy Carlton, Ruaidhrí Carmody, Sebastien Carpentier, Pablo Carravilla, Rebecca Carrier, Tyler Carrier, Nestor Carrillo, Victor Carrion, Alan Cartmell, Ana Luisa Carvalho, Danilo O. Carvalho, Patrizia Casaccia, Amelia Casamassimi, Alejandro Casas, José Casas, Jordan Casey, Patricia Cassina, Benoît Castandet, Carlos A. Castañeda, Pau Castel, Immacolata Castellano, Ainara Castellanos-Rubio, Jose R. Caston, Stefan Catheline, Lauren Cator, Isabella I. Cattadori, James F. Cavanagh, Patrick Cavanagh, Simona Ceccarelli, Violetta Cecchetti, Thomas R. Cech, Cristina Cereda, Biancastella Cereser, David Cerny, Camilla Cerutti, Carlo Cervia, Fabrizia Cesca, Igor Cestari, Jenu Chacko, Mohamed Chahine, Apirat Chaikuad, Maria Chait, Pimchai Chaiyen, Oishee Chakrabarti, Rumela Chakrabarti, Goutam Chakraborty, Romy Chakraborty

Dimple Chakravarty, Mallar Chakravarty, Dipshikha Chakravortty, Dan Challender, Janice Chambers, Jeanne Chambers, Joseph E. Chambers, Alex Ho Pang Chan, Conrad E.Z. Chan, Jo-Anne Chan, Kuan Rong Chan, Yujia Alina Chan, Natali Chanaday, Hitendra S. Chand, Navdeep S. Chandel, Anchal Chandra, Kasturi Chandra, Rajesh Chandramohanadas, Mahesh Chandrasekharan, Bo-Jui Chang, Jae-Byum Chang, Sunghoe Chang, Zee-Fen Chang, David B. Chapel, Eli Chapman, Manish Charan, Elena Chartoff, Alexander B Chase, Krishnananda Chattopadhyay, Dipayan Chaudhuri, Lise Chauveau, Amanpreet Singh Chawla, Frédéric Chédin, Alain Chedotal, Umber Cheema, Rosa M. Chefaoui, Martha Chekenya, Albert Chen, Ann Chen, Bing Chen, Changchun Chen, Chi Chen, Chongyi Chen, Chunying Chen, Congying Chen, Dong-bao Chen, Fadi Chen, Gang Chen, Hong-Hwa Chen, Huaiyong Chen, Huanhuan Joyce Chen, Hungwen Chen, Jeannie Chen, Jerry L. Chen, Jiahui Chen, Jichao Chen, Junjie Chen, Li Chen, Liang Chen, Ling Chen, Lu Chen, Mei-Kuang Chen, Min Chen, Ming-Fong Chen, Pao-Yang Chen, Peilin Chen, Peng Chen, Rui Chen, Sheng Chen, Wei Chen, Weiqiang Chen, Xian-Ming Chen, Xiaoyin Chen, Yan Chen, Yang Chen, Yolanda Chen, Yong Chen, Yu Chen, Yupeng Chen, Zhilei Chen, Zhuang-Gui Chen, Zhucheng Chen, Zi Chen, Zigui Chen, Benoit Chenais, Hao Cheng, Lily Cheng, Ping Cheng, **Xiaodong Cheng [4]**, David Cheresh, Leonid V. Chernomordik, Fabienne Chevance, Jeannie Chew, Meenu Chhabra, Chuan Chiang-Ni, Marcello Chieppa, Lounes Chikhi, Diana W. Chin, ShiNung Ching, E.Antonio Chiocca

Gabriela Chiosis, Kenny Chitcholtan, Hua-Sheng Chiu, Weihsueh A. Chiu, Carol Cho, Dong Seong Cho, Dong-Hyung Cho, Nam-Joon Cho, Seung-Woo Cho, Sung-Jin Cho, Sung-Joon Cho, You-Hee Cho, Fow-Sen Choa, Peter Choi, Shing Wan Choi, Methawi Chomthong, Siew Woh Choo, Ching-Chieh Chou, Salem CHOUAIB, Shantanu Chowdhury, Sourav Chowdhury, Kimberly M Christian, Merz J. Christian, Julian Christians, Lasse Christiansen, Michelle Christie, Kendra Chritz, Zofia M. Chrzanowska-Lightowlers, Chengjin Chu, Dinh Toi Chu, Hsueh-Ping Chu, Li-An Chu, Ying-Hsia Chu, Kwang-Hoon Chun, Hee Jung Chung, Henry Chung, Ka Young Chung, Won-Suk Chung, Jessica Church, Michael Church, Kristina Chyn, Marcus T. Cicerone, Radoslaw M. Cichy, John A. Cidlowski, Gözde Çilingir, Berta Cilleropastor, Gino Cingolani, Bruce Citron, Francesca Citron, Manfred Claassen, Nico J. Claassens, Emily Clark, Theodore Clark, Gerard Clarke, Michael Cleary, Timothy P. Cleland, Rollie Clem, Ann Clemens, Derek Clements, Nicolas Clemons, Roger Close, Gemma Clucas, Melanie Cocco, Dawn Cochrane, Emanuele Cocucci, Franca Codazzi, Enrico Coen, Rodrigo Cofre, **Richard J. Cogdell [4]**, Michael Cohanpour, Michael S. Cohen, Thomas Colgan, Remy Colin, Matthew Collin, Rebecca Colman, Luca Colnaghi, Giorgio Colombo, Karen Colwill, Lorenza Colzato, Erika Comasco, Simon Comerma, Mariana Conceicao, Fabien L. Condamine, Ana Conesa, Michaela J. Conley, Brian P. Conlon, Jan Conn, Charles E. Connor, Chris Conroy, Todd Constable, Christos Constantinidis, Roberto Contestabile, Douglas R. Cook, Martha Cook, Samantha Cook, Samuel Cooke, Mark Cookson, Mark Cooper, Isabelle Coppens, Cyril Corbet

Ryan Corbett, Laura J. Corbin, M R. Corces, Nils Cordes, Cinzia Corinaldesi, Richard Corlett, Yann Cormerais, Brian Corneil, Pierre Cornelis, Robert Cornell, Stephanie M. Correa, Melanie Correll, Andrew D. Corso, Aitziber Cortajarena, Alexandre Corthay, Ignasi Cos Aguilera, Mehmet Ilyas Cosacak, Ahmet F. Coskun, Diogo Alpuim Costa, **Irene Costantini [4]**, Cait Costello, Javier Cotignola, Chris Cotsapas, Elena Couce, Jennifer Coull, Elizabeth Coulthard, Pascal Coumailleau, David Courtney, Bruno Coutard, Felipe H. Coutinho, Daniel Coutu, Christine Couturier, Peter V. Coveney, Caitlin Cowan, James Cowan, Dominique Cowart, Mark Cowley, Dianne Cox, Philip Cox, Anthony G. Coyne, Mark Cragg, Brian R. Crane, Kerri Crawford, Meli’sa Crawford, Jacqueline Crawley, Emma Creagh, Jennifer Crees, Henk Cremers, Vinciane Saint Criq, Simona Cristea, Elizabeth Crofton, John E. Cronan, Rachelle H. Crosbie-Watson, Rebecca Cross, Anto Sam Crosslee, Michael Douglas Crowther, Eric Crubézy, John F. Cryan, Antonio Cuadrado, Francisco A. Cubillos, Maré Cudic, Luis Cuello, Alejandro Cueva, Jianmin Cui, Julia Yue Cui, Stephan Culemann, Kathryn A. Cunningham, Kyle Cunningham, Lorenzo Cupellini, Lukasz Cwiklik, Jason Cyster, Manoranjan D’Souza, Gisela D’Angelo, Pier Paolo D’Avino, Helton Da Costa Santiago, Luciana Jesus Da Costa, Alexessander Da Silva Couto Alves, Gabriela Da Silva Xavier, Marcel Daadi, Nabil Daddaoua, Ilse S. Daehn, Gerhard Dahl, Jan-Ulrik Dahl, Lukas R. Dahlin, Junbiao Dai, Lei Dai, Brad Daigneault, Sakano Daisuke, Alma Dal Co, Zachary Dalebroux, Pauliina Damdimopoulou, **Julia Dancourt [4]**, Erik Danen, Bobo Dang, Hien Dang, Rachel Daniels, Tal Danino, Davide Danovi, Tiago Dantas, Irina Dapic

Leonardo Dapporto, Frederic Darios, Bappa Das, Kinjal Dasbiswas, Vangelis Daskalakis, Thomas Daubon, Ana Daugherty, Fabian Davamani, Laurent David, Tom B Davidson, Daniel Davis, Felicity Davis, Gail Davis, Chi Ping Day, Felix R. Day, Caterina De Bacco, Marta De Barba, Wouter De Coster, Marcus C. De Goffau, Johannes De Jong, Tania De Koning-Ward, Susana De la Luna, Sara De la Salle, Bas De Laat, Silmara De Lima, Victor De Lorenzo, Daniele De Luca, Bruno A. De Medeiros, Delphine De Moor, Markus De Raad, Mariangela De Robertis, Cor De Wit, Joris De Wit, Chris G. Dealwis, Rashid Deane, Margaret DeAngelis, Tara L. Deans, Dominique Debanne, William DeBello, Frédéric Debiève, Alexiane Decout, Peter Dedecker, Bridget R. Deemer, Michael DeGeorgio, Ufuk Degirmenci, Kirk W. Deitsch, Borislav Dejanovic, Nava Dekel, Javier Del Pino, Ricardo Del Rosario, Andrew Delamater, Anne Delany, Luis Delaye, Michela Deleidi, Lucie Delemotte, Kaspar Delhey, Stefania Dell’Orso, Tonya DelSonto, Sofie Demeyer, Elizabeth Demicco, Christopher E. Dempsey, Tanneke Den Blaauwen, Hong-Wen Deng, Lih Wen Deng, Christine Dengler-Crish, Gina M. DeNicola, John J. Dennehy, Laura Denney, Robert J. Denver, Pankaj Deo, David Depew, Emily Derbyshire, Marta Derecka, Eske Derks, Philippe Derreumaux, Sanjay Desai, Ambroise Desfosses, David DeShazer, Antonio Desimone, Michèle Desjardins, Claire C. Detrain, Michael Deture, Jan Deussing, Ben Deverman, Didier Devys, James Dewey, Arti Dhar, Prarthana S. Dharampal, Guha Dharmarajan, Sirano Dhe-Paganon, Italia Di Liegro, Daniele Di Marino, Barbara Di Ventura, Dolores Di Vizio, Xin Di, Jean-Simon Diallo, Brian Dias, Bruno Díaz López, Ignacio Díaz Martínez, Ignacio Diaz-Franulic

Irene Díaz-Moreno, Fernando Diaz, Mani Diba, Robert P. Dickson, Anne Didier, Francesco Dieli, Katja Dierking, Sarah D. Diermeier, Gilles Dietrich, Alexandra G. DiFeliceantonio, Gareth Difford, Paul Dijkstra, Vishnu Dileep, Peter Dillingham, Stavros I. Dimitriadis, Rumiana Dimova, Astra Dinculescu, Hui Ding, Junjun Ding, Qiurong Ding, Xi-Qin Ding, Yousong Ding, Albena T. Dinkova-Kostova, Luisa Ann Dipietro, Naomi Dirckx, Larry Dishaw, Thomas Dittmar, Christina Dixelius, Rajnikant Dixit, Matthew Dixon, Mohammed Diykh, Mjgan Djavaheri-Mergny, Alexandre Djiane, Ana Djukovic, Jacek P. Dmochowski, Ross Dobie, Kostantin Dobrenis, F Stephen Dobson, Dylan Dodd, Hans-Ulrich Dodt, Thorsten Doeppner, Angelika Doetzlhofer, Priyanka Dogra, Tatsuya Dokoshi, Patrick T. Dolan, David Doležel, Philippe Domenech, Javier Dominguez Zamorra, Elisa Donato, Jin Dong-Yan, Haohao Dong, Xiaofeng Dong, Ryan Donnelly, Sandra Donnini, Jonathan S. Dordick, Zhicheng Dou, David A. Dougan, Adam Douglass, Linda Douw, Tim Downing, Nancy Doyle, Jeremy A. Draghi, Olga Dragoy, Allison Drain, Lika Drakhlis, Chris Draper-joyce, Hannah Drescher, Mikhail Drobizhev, Martin Drucker, Micha Drukker, Jianzhong Du, Xiangjun Du, Zhuo Du, Liting Duan, Zhaojun Duan, João Duarte, Danielle H. Dube, Mikhail Dubinin, Marcus Duchler, Vikas Dudeja, Iain G. Duggin, Thomas Duhen, Jorge Duitama, Jennifer Dulin, Guillaume Dumas, Andra C. Dumitru, Dominique Duncan, Gregg Duncan, Stephen Duncan, Laura Duncanson, Jessica Dunleavy, Mary J. Dunlop, Sirio Dupont, Joan Duprez, Leonardo Durante, Andrés Durantini, Thomas Durek, Aurélie Dutour, Heverton Dutra, Amit Dutt

Somnath Dutta, Craig Duvall, Zdenek Dvorak, Michael A. Dyer, David Dyment, Lars Dyrskjot Andersen, Helen J. Dyson, Nicolas Dzamko, Sarah Earle, Obialunanma V. Ebenebe, Hiromichi Ebi, Gloria V. Echeverria, Kristin L. Eckel-Mahan, Chandrakanth Edamakanti, Richard A. Edden, Matthew Eddy, Bradley Edelman, Frances Edwards, Martin Edwards, Thomas Edwards, Jorine Eeftens, Robert Eferl, Igor R. Efimov, Rouslan Efremov, Mirjana Efremova, Gregory Ehx, Pieter Eichhorn, Brandt F. Eichman, William Eimer, Judith S. Eisen, Jesper Eisfeldt, Karin Eisinger, Weronica E. Ek, Ahmet Eken, Moses Effiong Ekpenyong, Nady El Hajj, Marie-Therese El-Daher, Wael El-Nachef, Sam El-Osta, Shokrollah Elahi, James T. Elder, Coen Elemans, Leonardo Elia, Natalie Elia, George Elias, Moshe Elkabets, Eyad Elkord, Hannah Elliott, Kerryn Elliott, Georgina Ellison-Hughes, Wilfried Ellmeier, Khaled Elokely, Simon Elsässer, Hany M. Elsheikha, Amira M. Eltony, Sara Emery, Uzay Emir, Edward Emmott, Steven Emslie, **Sandra E. Encalada [4]**, Jörg Enderlein, Iuliana Ene, Charis Eng, Martin M. Engelhard, Benjamin Enger, Martin Engqvist, Giray Enkavi, Soo Hyun Eom, Jesse Erasmus, Aaron Ericsson, Jason Ernst, Claire Eschbach, José Escudero, Mitra Esfandiarei, Camila Esguerra, Dani Eshel, Changiz Eslahchi, Maria Rosaria Esposito, Oscar Esteban, Iryna M. Ethell, Anders Etzerodt, Philipp Euskirchen, Sebastian Eustermann, Eric Evans, Jay D. Evans, Louise C. Evans, Damien Eveillard, Seong-il Eyun, Teresa Ezponda, Matteo Fabbri, Cornelius Faber, Emmanuelle Fabre, **Federico Pio Fabrizio [4]**, Elisa Fadda, Christoph Fahlke, Donald Falk, Luca Falzone, Pengxiang Fan, Songtao Fan, Wei Fan

Marcus Fändrich, Gang Fang, Rongxin Fang, Tilin Fang, Yue Fang, Katherine A. Fantauzzo, Frank M. Faraci, Ibrahim Farag, Hesso Farhan, Henner F. Farin, Carlos M. Farinha, Reinhard Fässler, Robert Faure, Denise Faustman, Herman W. Favoreel, Colin Favret, **Mark Fear [4]**, David Fedida, Brad Fedy, Zhangjun Fei, Michael Feigin, Christoph Feinauer, Michal Feldman, Sebastien A. Felt, Xiao Feng, Xizeng Feng, Youzhi Feng, Zhiyang Feng, Ziang Feng, Leif Fenno, Brock M. Fenton, Jean-Luc Ferat, Farzad Fereidouni, Jane F. Ferguson, Michael A. Ferguson, Scott Ferguson, Stuart Ferguson, Carlos Fernandez-Patron, Erika Fernandez-Vizarra, Luis Angel Fernandez, Rosa Fernandez, Alisdair Fernie, Maria Immacolata Ferrante, Fortunato Ferrara, Aldo Ferrari, Pietro Ferraro, Joao N. Ferreira, Donna Ferriero, Soldano Ferrone, Marco Festa-Bianchet, Franzi Fichtner, Konrad Fiedler, Mark Field, Edward Fielder, Andrew Fielding, William D Figg, Nikolay M. Filipov, Jessica Filosa, Iris Finkemeier, Sjoerd Finnema, Valentina Fiorilli, Mohd Firdaus-Raih, Carola Fischer-Tenhagen, Andy Fischer, Julia Fischer, Frank F. Fish, Lisa Fish, Charles Fisher, Sylvia Fitting, Elizabeth Fitzpatrick, Kathryn Fixen, Shelly Flagel, Sarah Flanagan, Meghan Flanigan, Jason A. Flannick, Kyle Fletcher, Veit Flockerzi, Cecilia Flores, Gilberto Flores, Oliver Florey, Bernhard Flucher, Regina Fluhrer, Andrew Flyak, Kevin Flynn, Daniele Focosi, Ervin Fodor, John Foerster, Hana Foffova, Fatima Foflonker, Sylvain Foissac, Saoirse Foley, Matilde Follo, Thomas Foltynie, Yick Fong, Joel Fontaine, Juan Fontana, Laura Fontanesi, Silvia Fontenete, Suan-Sin Foo, Judith M. Ford

**Stephanie Forkel [5]**, Myriam Fornage, Matteo Fornai, Robert Foronjy, Lars A. Forsberg, Patrick Forterre, Mehrnush Forutan, Jade K. Forwood, Silvia Fossati, Daniel Foster, Simon Foster, **Chris Foulon [4]**, François Foulquier, Amanda Foust, Stephanie L. Fowler, David Fox, Enric Frago, Nora Franceschini, Charles Francis, Claudio A. Franco, Luis M Franco, Jakob Franke, Laurent Frantz, Alexander W E Franz, Henrik Franzyk, Danielle Fraser, James S. Fraser, Marie E Fraser, **Stefan Frässle [4]**, David Frazer, John K. Frazer, Cody Freas, Brian Fredensborg, Brian Freeman, Mary C. Freeman, Spencer A. Freeman, Alexander Fremier, Celine H. Frere, Natalia Freund, Christoph Freyer, Florian Fricke, Rick Friedman, Patrick Friedrich, Giovanni Frighetto, Daniel E. Frigo, Christian Frings, Friedrich Frischknecht, Megan Fritz, Eleonore Fröhlich, Tobias Fromme, Karl-Heinz Frommolt, Martin Fronius, Florian Frugier, Marcus Fruttiger, Mark Frye, Chuanhai Fu, Dan Fu, Peiliang Fu, Xianghui Fu, Yang Fu, Jitka Fucikova, Gabriel Fuente-Gomez, Gustavo Fuertes, Nobuto Fukuda, Ryuya Fukunaga, Ben D. Fulcher, Helena Fulka, David D. Fuller, Natalia Fumagalli, Hironori Funabiki, Katsuhiko Funai, Susanne Aileen Funke, Seth Furgeson, Yusuke Furukawa, Chikara Furusawa, Diana Fusco, Monika Fuxreiter, Gisela Gabernet, Ella Gabitov, Hans-Joachim Gabius, Gülsah Gabriel, Paolo Gabrieli, Frederic Gachon, Jennifer A. Gaddy, Bart Gadella, Paul Gadue, Carlo Gaetano, Nicola Gagliani, Teresa Gagliano, Karine Gagnon, Alexander Gail, Michelle Gaither, Thomas Gaj, Takashi Gakuhari, Péter Galajda, Francisco X. Galdos, Gabriel L. Galea, Mario D. Galigniana, Roberto Galizi, Alexander Galkin

David Gallacher, Nathan Gallant, Cécile Gallea, Pedro P. Gallego, Giuseppe Galletti, Thierry Galli, Jenna Gallie, Fabiane Gallucci, Ralf Galuske, Ismael Galván, Laurie Galvan, Leonardo J. Galvão-Lima, Benoît Gamain, Doaa Gamal, Alicia Gamboa-deBuena, Jianhua Gan, Uday Ganapathy, Amit Gandhi, Karuna Ganesh, Sreeramaiah N Gangappa, Karunesh Ganguly, Maureen Gannon, Markus Ganter, Dongying Gao, Jianzhao Gao, Rong Gao, Zhan-Guo Gao, Gyozo Garab, Michael J. Garabedian, Silvia Garavaglia, Juan Garaycoechea, Ruben Garcia-Cabrerizo, Francisco Garcia-Del Portillo, Paula Garcia-Fraile, Ricardo Garcia, David Gardner, Eugene J. Gardner, Venkata Garikipati, George G. Garinis, Dorian Garrick, Danielle Garsin, Fátima Gärtner, Zev J Gartner, Josep M. Gasol, Nicholas Gaspelin, Valeria Gasperi, Susan M. Gasser, Allison Gaudinier, Patricia Gaule, Jeffrey Gauthier, Arnaud Gautier, Christina Gavegnano, Leonid A. Gavrilov, Kinga Gawel, Marcos Leoni Gazarini, Valeria Gazzola, Xing-Yi Ge, Yubin Ge, Zhengpeng Ge, Ester Gea-Mallorquí, Grum Gebreyesus, Danny Geelen, Eldon Geisert, Edward Geisinger, Werner Geldenhuys, Neil J. Gemmell, Louis Gendron, Andrea Genre, Steve Gentleman, Geraldine Gentric, Rebecca Gentry, Lisa Genzel, Julia George, Julie George, Olivier George, Sally L. George, Senta K. Georgia, George Georgiou, Anthony Gerber, Christoph Gerhardt, Daniel W. Gerlich, Michael Germuska, Moritz Gerstung, Jason Gertz, Klaus Gerwert, Dixon Gevaña, Matthias Geyer, Soosan Ghazizadeh, Roberto Gherzi, Reena Ghildyal, Barbara Ghinassi, Fabrizio Ghiselli, Mahdi Ghorbani, Debashree Ghosh, Mallika Ghosh, Stefania Giacomello, Natasa Giallourou, Georgios Giamas, Daniel Gianola, William J. Giardino

Luis Giavedoni, Theo Gibbs, Lara Gibellini, Erin Gibson, David P. Giedroc, Lila M. Gierasch, Melissa Gilbert-Ross, Christopher Gilbert, Luke Gilbert, Mark R. Gilbert, Jeffrey Gildersleeve, Todd Gilligan, Paul R. Gilson, Daniel Gioeli, Facundo Giorello, Manuela Giovannetti, Katta M. Girisha, Sylvain Giroud, Daniel Gitler, Jimena Giudice, Brian Giunta, Giorgio Giurato, Martin Giurfa, David Gius, Enrico Glaab, Ian Glaaser, Candece L Gladson, Jonathan Glass, Dominique A. Glauser, Sarah Glaven, Andy Gloss, Hervé Glotin, Anna L. Gloyn, Selene Gluck, Steven E. Glynn, Geoffrey Gobert, Keisuke Goda, Ben Godde, Ramiro Godoy-Diana, Christian Goeritz, Gergő Gógl, Michel Gohar, Ivana Gojo, Nandan Gokhale, Matan Golan, Michael R. Gold, Jesse H. Goldberg, Alisa Goldstein, Stephen Goldstein, Ewa Goldys, Erin D. Goley, Leonardo Gollo, Kandace Gollomp, Mário Gomes-Pereira, Maria Victoria Gomez-Gaviro, Elisa Gomez-Perdiguero, Ludvik M. Gomulski, Hui Gong, Ming Cui Gong, **Wei Gong [4]**, Concepción González Bello, Sara Gonzalez-Arranz, Christian Gonzalez-Billault, Jordi Gonzalez-Molina, Daniel Gonzalez, Ramón González, Reid Gooch, Cynthia Goodman, Jeffrey Goodman, Nastacia Goodwin, Paul R. Gooley, Ellen Goossens, Smita Gopinath, Isabel Gordo, David J. Gordon, Michael D. Gordon, Reuven Gordon, Reyna Gordon, Renee V. Goreham, Vassilis G. Gorgoulis, Kevin Gori, Natalie K. Goto, Taichiro Goto, Nicole Gottdenker, Joel M. Gottesfeld, Claudia Göttsch, Benjamin Goudey, Alexandros Goulas, Dennis Goulet, Marie José Goumans, Eleni Gourgou, Samudrala Gourinath, Geoffrey Gourinchas, Mateus Gouveia, Alex Gouzy, David Goyer, Fabian Grabenhorst, Anna Grabowska, Elena Gracheva, Rebecca E. Graff

Sheila Graham, Victoria Graham, James Granneman, Nkrumah Grant, Susanne Grässel, Julia Grassl, Dominique Gravel, Alison Grazioli, Erin Greaves, Alistair Green, Shawn Green, **Maxim Greenberg [4]**, Matt Greenblatt, Casey S. Greene, Andrew Greenhalgh, Elisa Greggio, Anna Greka, Christina Gremel, Melanie Greter, Michelle Greve, Christof Grewer, Blaine Griffen, Amy Griffin, Courtney Griffin, Oliver W. Griffith, Howard Griffiths, Matthew J. Grigg, Ramon Grima, Daniel Grimanelli, Simone Grimm, Andrew Grimson, Alla Grishok, Simon Groen, Natalia Gromak, Aaron Gross, Alecia K. Gross, Christina Gross, Richard Gross, Aaron Grossberg, Madhusudan Grover, Edward Grow, Zorana Grubic, Kristina Gruden, Benjamin F. Gruenbaum, Ireneusz Grulkowski, Alexei Gruverman, Xiaofeng Gu, Maëlle Gueguen, Yann Guerardel, Dr. Marcelo E. Guerin, Geraldine Gueron, Michele Guescini, Fu Gui, Cynthia J. Guidos, Sonia Guil, Karen Guillemin, Julie Guillermet-Guibert, Caitriona Guinane, Claire Guinat, Rao Gullapalli, Ozan Gundogdu, Bartu E. Güneşliol, John Gunn, Eoin Gunnigle, Chengchen Guo, Fukun Guo, Ju-Tao Guo, Wenjun Guo, Xiaonan Guo, Vikas Gupta, Shiri Gur-Cohen, Hakki Gurhan, Güray Gürkan, Katerina Gurova, Jose F. Gutierrez-Marcos, Alejandro Gutierrez, Andrew Gutierrez, Vishwesha Guttal, Segundo J. Guzman, Ulf Gyllensten, David Gyori, Jan Haaker, Thomas R. Haaland, Astrid D. Haase, Hajo Haase, Benjamin Hackel, Hubert Hackl, David Hackman, Wilfried Haerty, Ziad M. Hafed, Melanie Haffner-Luntzer, Iva Hafner‐Bratkovič, Peter-Leon Hagedoorn, Andreas Hahn, Daniel Hahn, Heidi Hahn, William C. Hahn, Sarah Haigh, Niina Haiminen, Norbert Hajos

Pierre Hakizimana, Christopher J. Halbrook, Malay Haldar, Shubhasis Haldar, Georg Halder, Tracy Hale, Ian Hall, Margaret Hall, Mary Halloran, Michelle L. Halls, Peter Hamilton, Sara Hamilton, Kamel Hammani, Michal Hammel, Chrissy L. Hammond, Gerald (R.V.) Hammond, Byung Woo Han, Chao Han, Dong-Wook Han, Hyunho Han, Jae Yong Han, Xue Han, Yi Han, Abdulsamie Hanano, Adam Handel, Mary Ann Handel, Stephanie Hanna, Anthony Hannan, Nicholas (RF) Hannan, Gregory J. Hannon, E. Hannula, Axel K. Hansen, Christian Hansen, Immo Hansen, Oliver Hantschel, Goro Hanya, Xingjie Hao, Alexandre Harari, Oscar Harari, Ben Hardcastle, Itamar Harel, Ahmad Hariri, Victoria S. Haritos, Takahiko Hariyama, Arif Harmanci, Stephanie Harmon, Elizabeth Harper, Andrzej Harris, Ed Harris, Ian Harrison, Oliver Harrison, Stephen C. Harrison, Ulrich Hartl, Erica M. Hartmann, Rachel Hartnett, Robert J. Harvey, John Harwood, Saif Hasan, Masato Hasegawa, David Haselbach, Kazuhide Hashiya, Martina Hason, Jasmin Hassan, Helen Hathaway, Alexander Hatoum, Mark E. Hauber, Volker Haucke, Peter Havel, Shlomo Havlin, Cheryl Hawkes, Nichola Hawkins, Davie Hawksworth, Kanako Hayashi, Katsuhiko Hayashi, Tetsuya Hayashi, Yohei Hayashi, Khaled M. Hazzouri, Biyu J. He, Changjiu He, Kaiwen He, Mingguang He, Qixin He, Jason Head, Adam L. Healey, Luke Hearne, Elizabeth Heath-Heckman, George Heath, Victoria Heath, Terence Hébert, Nicola Heckeberg, Nikolai Hecker, Brandon Hedrick, Csaba Hegedűs, Tamas Hegedus, Thies Marten Heick, Johann Heider, George E Heimpel, Andreas Heine, Julie Heinrichs, Peter D. Heintzman

Sophie Helaine, Marcus Heldmann, Simon Hellemans, Sarah Hellewell, Ute A. Hellmich, Thomas Hellwig-Bürgel, Andrew Hemphill, Marcela Henao-Tamayo, Chew-Kiat Heng, Rob Henning, Michael Henshaw, Richard N. Henson, Sian Henson, Yann Herault, Guillaume Herbet, Ruth Herbst, Timothy R. Hercus, Enrique Hernández-Lemus, Maria Hernandez-Valladares, Erick Hernandez, Ingrid Herr, Edgar Herrera-Delgado, Carolina Herrera, Javier Herrero, Michael Herrmann, Maria Herrojo-Ruiz, Pamela A. Hershberger, Uri Hertz, Cecile Hervé, Ralf Herwig, Geoffrey Hesketh, Jochen Hess, Robert F. Hess, Daniel Hesselson, Joanna Hester, Mark E. Hester, Rainer Heuchel, Mark Hicar, Clayton Hickey, Shigeaki Hida, Cecilia Hidalgo, Falk Hildebrand, Sven Hildebrand, Elisa L Hill-Yardin, Michelle M. Hill, Steve Hillyard, Julian Hillyer, William Hines, Christian Hintze, **Yusuke Hirabayashi [4]**, Katsuya Hirano, Makoto Hiroi, Alec Hirsch, Liisa Hirvonen, Peter Hitchcock, Richard Hite, Carl Hjelmen, Jens Hjerling-Leffler, Meng-Chiao Ho, Robin M. Hobbs, Chad Hobson, Dorothee Hodapp, Guillaume Hoeffel, Nolte-‘t Hoen, Andrew Hoey, Ary Hoffmann, Markus Hoffmann, Thomas J. Hoffmann, Kay Hofmann, Maike Hofmann, Zuzana Hofmanova, Michael Hofreiter, Paulo G Hofstatter, Shir Hofstetter, Alison Hogan, Julia Höglund, Mateusz Hohol, Erling A. Hoivik, Michael Holbrook, Séamus Holden, Peter W. Holland, Frédéric Hollande, Edward J. Hollox, Andrei Holodny, Nicholas Holowka, Hendrik Hölscher, Daniela Holtgräwe, Marina Holz, Evert Homan, Charles C. Hong, Lu Hong, Mei Hong, Tian Hong, Christer Höög, Gareth Hopkins, Adam D. Hoppe, Mathias W. Hornef, Eric Horstick, Atsushi Hoshino, Ayuko Hoshino

Kazunori Hoshino, Samira Hosseini-Alghaderi, Akitsu Hotta, Hongwei Hou, Wenpin Hou, Zhuo-Cheng Hou, Russ Hovey, Laurence Howe, Leann J. Howe, Kate Howell, Gerta Hoxhaj, Irene Hoxie, Wolfgang Hoyer, Marc Hoylaerts, Connie Hsia, Shannon Hsieh, Shie-Liang Hsieh, Amy Hsu, Hwei-Jan Hsu, Shang-Te Danny Hsu, David Hu, Kejin Hu, Ping Hu, Shi Hu, Song Hu, Wensi Hu, Xiaoping Hu, Yanling Hu, Yibo Hu, Chung-Ching Hua, Chuanxin Huang, Haojie Huang, Heng Huang, Jie-rong Huang, Lulin Huang, Suyun Huang, Tao Huang, Ting-Ting Huang, Y Mindy Huang, Yu Huang, Yubin Huang, Zirui Huang, Ziwei Huang, Jochen S. Hub, Eva Huber, Gregory A. Hudalla, Robert Huff, Matthew B. Hufford, Clemens Hug, Terry P. Hughes, Willy Hugo, Cang Hui, Jie Hui, Mark O. Huising, Laura Hulea, Mahmoud Huleihel, Patrick O. Humbert, Emily Humble, Mary B. Humphrey, Mark Humphries, Rayjean Hung, David M Hunt, Johannes B. Huppa, Jenniver Hurley, Holger Husi, Mushtaq Hussain, Michael Hust, Joshua Hutcheson, Andrew P Hutchins, Dana S. Hutchinson, Alexander Huth, Carl Hutter, Malthe Hvas, Torgeir R. Hvidsten, **Wendy Hwang-Verslues [4]**, Nathaniel Hwang, Kelly Hyndman, Christian Ibáñez, Hidenori Ichijo, Ronaldo Ichiyama, Konstantin Ichtchenko, Alexander Idnurm, Akira Iguchi, Masaya Ikegawa, Taichiro Iki, Subburaj Ilangumaran Ilangumaran, Zehra Esra Ilhan, Patricia T Illing, Yoshikazu Imanishi, Anthony N. Imbalzano, Anne Imberty, I. Imoukhuede, Francis Impens, Francesco Imperi, Noriko Inada, Eugenia Inda, Alyssa Ingmundson, Asuka Inoue, Satoshi Inoue, Yasuhiro Inoue

Janis Intoy, Andrea Introini, Thoman Ioerger, Alexandru Iordan, Juan L. Iovanna, Patricia Iozzo, Manuel Irimia, James Irving, Tadashi Isa, Krista Isaacs, Ehud Y. Isacoff, Akira Ishihama, Nina Isoherranen, Kiyoshi Itagaki, Etsuro Ito, Yutaka Ito, Sirawaj (Sean) Itthipuripat, Alexander G. Ivanov, Andrei Ivanov, Andrey A Ivanov, Plamen C. Ivanov, Anna Ivanova, Edward Ivimey-Cook, Sarah Ivory, Jess Ivy, Hideo Iwaï, Ravi Iyengar, Smita Iyer, Vivek Iyer, Tina Izard, Abril Izquierdo, William Ja, Dick Jaarsma, David Jackson, Fatimah L.C. Jackson, Katherine J.L. Jackson, Michele Jacob, Joshua Jacobs, Suzanne B R Jacobs, Guillaume Jacquemet, Ramesh S. Jadi, Cho Jae-Hyun, Jean-Jacques Jaeger, Amirhossein Jafarian, Ali Jahanshahi, Roman Jakob, Arjen Jakobi, Stephen Jamieson, Daniel Janies, Mark Jankauski, Jakub Jankowski, Harald Janovjak, Robert Jansen, Joanne Jao, Li-En Jao, Simon Jarman, Timothy J. Jarome, Jessie Jarvis, Lorna B. Jarvis, Parmjit Jat, Jean-michel Jault, Marie-Claude Jaurand, Jean Paul Javerzat, Jonathan A. Javitch, Daniel Jeffares, Kate J. Jeffery, Robert Jehle, Nico Jehmlich, Andreas Jekle, Albert Jeltsch, Victoria Jennings, Charlie Jennison, Karin Jensen, Mogens Jensen, Thomas Jensen, Cherlhyun Jeong, Se-Jin Jeong, Roman Jerala, Marc G. Jeschke, Thomas Jetton, Anahid Jewett, Suman Jha, Boyang Ji, Minbiao Ji, Xiao-Jun Ji, Haibo Jiang, Hua Jiang, Huawei Jiang, Jian-Hui Jiang, Rui Jiang, Shibo Jiang, Yan Jiang, Yong-Hui Jiang, Yuchaoj Jiang, Zhilong Jiang, Yang Jiao, Zongxian Jiao, Abdulqader Jighly, Jose M. Jimenez-Guardeño, Biao Jin

Jianping Jin, Victor Jin, Xiaoming Jin, Zhe-Wu Jin, Naihe Jing, Young-Hwan Jo, Stephanie Joachim, Carine Joffre, Frank Johannes, Jan Johansson, Friedrich Johenning, Lily Johnson-Ulrich, Andrew Johnson, **Gregory A. Johnson [4]**, Kevin C. Johnson, Kirby Johnson, Rory Johnson, Winifred Johnson, Alice Johnston, Elizabeth K. Johnstone, Marc Joliot, Elizabeth Jones, Jeff Jones, Katrina Jones, Lesley Jones, Nikoleta Jones, Gregory Jongsma, King Jordan, Mehdi Jorfi, Rakesh Joshi, Daniel Jost, Laurent Journot, Jovana Jovičić, Christopher Joy Mathew, Tingting Ju, Esmeralda Juárez, Gabor Juhasz, Olivier Julien, Mi-Yeon Jung, Sang Taek Jung, Steffen Jung, Tae Sung Jung, Jan Philipp Junker, Virpi Junttila, Kellie Jurado, Irina Kabakova, Ryoichiro Kageyama, Barbara Kahl, Michelle Kahlenberg, Samuel Kahng, **Kenji Kai [4]**, Toshie Kai, Taku Kaitsuka, Rebecca Kalinger, Vladimir Kalinichenko, Nir Kalisman, Amanda Kallen, Elisa Kallioniemi, Pawar Kalpana, Julia Kam, Tomoshi Kameda, Ken-ichiro Kamei, Amine Kamen, Laura Kamenetzky, Hironori Kaminaka, Tomasz S. Kaminski, Timothy Kamp, Harm H. Kampinga, Radhakrishnan Kanagaraj, Pakorn Kanchanawong, Eaazhisai Kandiah, Lawrence Kane, Bin Kang, Congbao Kang, Jeon Woong Kang, Un Jung Kang, Wei Kang, Zhensheng Kang, Stephen A. Kania, Katy C. Kao, Mimi Kao, Ronan Kapetanovic, Karen Kapheim, Gisela Kaplan, Mohit Kapoor, David Kappel, Reuben Kapur, Anastasios Karadimitris, Matthias Karajannis, Erkan Karakas, Narain Karedla, Michael Kareta, Russell Karls, Jan Karlseder, Vyacheslav Karolis, Ajith Karunarathne, Zam Kassiri, Ryohei Katayama, Ashley Kates, **Hideaki Kato [4]**

Liora Katz, B. Kaupp, Kamaljit Kaur, Akash Kaushik, Reika Kawabata-Iwakawa, Masayoshi Kawaguchi, Shigeyuki Kawai, Koichi Kawakami, Emily Kawaler, Hisashi Kawasaki, Toshimitsu Kawate, Lawrence Kazak, Negin Kazemian, James Kazura, Ailong Ke, Michael Kearney, Jay D. Keasling, Natalia Y. Kedishvili, Kehkooi Kee, Robert Keenan, Vladimir Kefalov, Hareshkumar Keharia, Benjamin Keitz, Andrew Kelleher, Andreas Keller, Sandro Keller, Matthew W. Kelley, Neil P. Kelley, Martin J Kelly, Caroline Kelsey, Gyorgy Kemenes, David Kemp, Michael Kemp, Roslyn Kemp, Genevieve Kendall, Candia M. Kenific, Toby Kenney, Carole Kerdelhué, Jan Kern, Tom K. Kerppola, Julie Kerr-Conte, Emma Kerr, Erin E. Kershaw, Kayvan Keshari, Nicoletta Kessaris, Amit Khairnar, Annette R Khaled, Walid T. Khaled, Ahmed Khalil, Abbas Khan, Fahim Halim Khan, Mohammad Moshahid Khan, Naim Akhtar Khan, Shahrzad Kharabian Masouleh, Michael G. Kharas, Joubert B. Kharlyngdoh, Roustem Khazipov, Alastair Khodabukusd, Kay-Hooi Khoo, Chiea Chuen Khor, Pegah Khosravi, Ekaterina E. Khrameeva, Konstantin Khrapko, Syed (Jalal) Khundmiri, Surender Khurana, Gregory Kiar, Michael A. Kiebish, Bruno Kieffer, Anna Kietrys, Eiki Kikuchi, Taisei Kikuchi, Chulhong Kim, Daehwan Kim, Dong Young Kim, Dong-Eun Kim, Hae Won Kim, Hong Kyun Kim, Hyongbum Henry Kim, In-Kwon Kim, Jae Bum Kim, Jae‐Hwan Kim, Junil Kim, Ki-Wook Kim, Leo Kim, Min-Ho Kim, Peter Kim, Pora Kim, Yongsoo Kim, Erin Kimbrel, Adi Kimchi, Jason Kindrachuk, Jace King, Jean-Rémi King, Samantha J. King, Paul Kingsley, R Manjunatha Kini, Akihiko Kinoshita, Lynne Kiorpes, Tod Kippin, Robert N. Kirchdoerfer

Elsa A. Kirchner, Devin Kirk, Leif Kirsebom, Yusuke Kishi, Joseph Kissil, Harold C. Kistler, Ryo Kitada, Gaspar Kitange, Johann P. Klare, Alexander Kleger, Miriam C. Klein-Flugge, David C. Klein, Elise Klein, Holger Klinck, Manfred Klöbl, Kathy Klos, Adam Klosin, Judith Klumperman, **Sandra S. Knapp [5]**, Vincent Knight-Schrijver, Maria J. Knol, Eileen Knorr, Emily Knott, Erik Knutsen, Dae Kwan Ko, Jaewon Ko, Hui-Fern Koay, Dmitry Kobak, Leonie Koban, Tatsuya Kobayashi, Jens Koblitz, Martina Kocan, Christian Koch, Sheryl Koch, Mattheos Koffas, Jun-ichiro Koga, Michael M. Kohl, Brooks Kohli, Samuel Kohtala, Tsuyoshi Koide, Ai Koizumi, Waldemar Kolanus, Evangelos Kolettas, Philip Kollmannsberger, Harald Kolmar, Jumpei Kondo, Roman Kondratov, Xiang-Zhen Kong, Talia Konkle, Roland E. Kontermann, Seung-Hoi Koo, Gijs Kooij, Sander Kooijman, Frank Kooy, Ryan Koppes, Konstantinos Kormas, Kseniya Korobchevskaya, Sergey Korolev, Nicole Koropatkin, Ron Korstanje, Keegan Korthauer, Jan Kosinski, Saori Kosono, Therese Kosten, Serhii Kostrikov, Christopher M. Koth, Ninad Kothari, Leah Kottyan, Fani Koukouli, Kostas Kousis, Marios Koutsakos, Christos Koutsarnakis, Gabriela Kovacikova, Ákos T Kovács, Ryuta Koyama, Dima Kozakov, Pawel Kozielewicz, Julia Kozlitina, Angela Krackhardt, Reuben H. Kraft, Charlotte Krahe, Rafael Kramann, Eva-Maria Krämer-Albers, Carolyn Kramer, Lee Kraus, Cory Krediet, Mateja Erdani Kreft, Hans-Jürgen Kreienkamp, Gabriel Kreiman, Jakub Kreisinger, Ute Krengel, Alena Kress, Michaela Kress, Tim Krischuns, Jaya Krishnan, Siddarth Krishnan, Peter Kristensen, Roland Kröger, Grzegorz Króliczak, Thorsten Krömer

Jeffrey Kroon, Jim Krueger, Christopher Krupenye, Shihuan Kuang, Fumi Kubo, Norbert Kucerka, Grzegorz Kudla, Yasusei Kudo, Misha Kudryashev, Irina Kufareva, Heiner Kuhl, Michael Kuhn, Eric Kuhnert, Jirko Kühnisch, Jonas Kuiper, Omar Kujan, Maria Kukley, George Kukolj, Tomasz Kula, Janesh Kumar, Jitendra Kumar, Manish Kumar, Prakash Kumar, Sharad Kumar, Vibhor Kumar, Dharshan Kumaran, Sonam Kumari, Takashi Kumasaka, Kazuhiko Kume, Grit Kunert, Yan Kung, Gary Kunkel, Annette Künkele, Lukas Kunz, Karl Kunzelmann, Alan Kuo, Shigehiro Kuraku, Morito Kurata, Mirian A. Kurauti, Jeff Kuret, Mariola Kurowska-Stolarska, Vartan Kurtcuoglu, Dennis Kurzbach, Sebla Kutluay, Lena Kutscher, Andrei Kuzminov, Kenneth Y. Kwan, Sze Ting (Cecilia) Kwan, Anne E Kwitek, Eun-Soo Kwon, Peter D. Kwong, Natasha Kyprianou, Vasileios Kyttaris, Mario La Mesa, David P. Labbé, David Labonte, Franziska Labrenz, Dimitris Lagos, Leo Lahti, Cora Sau Wan Lai, Dongbing Lai, Ren Lai, Partida-Martínez Laila, Romain F. Laine, Eszter Lakatos, Peter Lakatos, Jeremy Lakey, Klio Lakiotaki, Aparna Lakkaraju, Avantika Lal, Hon-Ming Lam, Deepak A. Lamba, Sander Lamballais, Jean-Philippe Lambert, Nevin A. Lambert, Urpo Lamminmäki, Shawn Lamothe, Valerie Lamour, Erwin Lamping, Maria Grazia Lampugnani, Ning Lan, Benjamin Land, Robert Landick, Christian R. Landry, Marene Landström, TJ Lane, Richard A. Lang, Stephan Lange, Benjamin Langmead, Gordon Langsley, Johanna Lanner, Caleb Lareau, Mioara Larion, Christopher LaRock, Doug LaRowe, Luis F. Larrondo, Erik Larsen, Melinda Larsen, Peter Larsen, H Peter Larsson

Michelle LaRue, **Paul Lasko [4]**, Kate Laskowski, Leena Latonen, Juan Latorre, Romain Lavaud, **Gregory Lavieu [5]**, Antonina Lavrentieva, Rubina Lawrence, Matthew B. Lawrenz, Devon A. Lawson, Michael Lawson, Olga Lazareva, Ronan Le Goffic, Duc (H.T.) Le, Weidong Le, Richard D. Leapman, Andrew Leask, Djamel Lebeche, Jeanne Leblond Chain, Guillaume Lebon, Stéphanie Lebreton, André Lechel, Beata Lecka-Czernik, Deborah Leckband, India Leclercq, Clotilde Lecrux, Marc Lecuit, Carsten W. Lederer, John Lednicky, Amy Lee, Andrea J Lee, Christopher Lee, Francis S. Lee, Insuk Lee, Jae-Hyeok Lee, Jae-Seong Lee, Jasmine R. Lee, Je Hyuk Lee, Jun-Seok Lee, Junmin Lee, Kyongbum Lee, Kyung-Woo Lee, Min Gyu Lee, Monica Lee, Norman Lee, Sang Hong Lee, Se-Jin Lee, Seung Hwan Lee, Sung Bae Lee, Yan Lee, Germain Lefevbre, Sjannie Lefevre, Julie Lefort, Roger Lefort, Wesley Legant, Brian D. Lehmann, Tovi Lehmann, Klaus Lehnertz, Mark Lehrman, Lei Lei, Mingxing Lei, James Leigh, Ed S. Lein, Rafael Fernandez Leiro, Victoria Leitch, Michael Leitges, Alexander Leithner, Birgit Leitinger, Franck Lejzerowicz, Ylva Lekberg, Raphaël Leman, Darrin Lemer, William Lemon, Christophe Lenglet, Suzanne Lentzsch, Maximilian Lenz, Tobias Lenz, Umberto Leon-Dominguez, Roberta Leonardi, Anna V Leopold, Brian Lerch, Frederik Lermyte, John F. Leslie, Joost Lesterhuis, Adrian Leuchtmann, Jeanette Leusen, Marcel Leutenegger, Sima Lev, Elena Levantini, Romain Levayer, Valeria Levi, Netta Levin, Herbert Levine, Joel D. Levine, Joshua Levitz, Anna-Liisa Levonen, **Monika Lewinska [5]**, Nathan E. Lewis, Patrick A. Lewis

Phillip Lewis, Simon Lewis, Zachary A. Lewis, Barbara Lewko, Luc Leybaert, Yonghua Li-Beisson, Aming Li, Bowei Li, Boxing Li, Chao Li, Chengdao Li, Chengqi Li, **Christine Li [4]**, Dahong Li, Duan Li, Fei Li, Fubin Li, Fuhai Li, Hong Li, Huilin Li, Ji Li, Jun Li, Lei Li, Lian-Yun Li, Lin Li, Lin-Feng Li, Mei Li, Meng-Hua Li, Ning Li, Peng Li, Qian Li, Sean Li, Shenghui Li, Shiguo Li, Song Li, Tuo Li, Wei Li, Wenbo Li, Xingnan Li, Xuri Li, Yanran Li, Yanyan Li, Yongle Li, Yuanfeng Li, Yun Li, Yunkai Li, Yusheng Li, Zhongwei Li, Zhongyu Li, Cui Liang, Rubing Liang, Yujie Liang, Evi Lianidou, Mingzhi Liao, Marc Libault, Maxime Liberelle, Assunta Liberti, Beate Lichtenberger, Jeffery T. Lichtenhan, Cheryl F. Lichti, Shane A. Liddelow, Thomas Liehr, Soeren Lienkamp, Marc Liesa, Sonia Liggi, Symen Ligthart, Chunghun Lim, Hui-Ying Lim, **Theam Soon Lim [4]**, Laurent Limozin, Audrey T. Lin, Chia-Hua Lin, Donghai Lin, Hanzhi Lin, Jia-Ren Lin, Michael Z. Lin, Xianhe Lin, Xiaorong Lin, Yu-Wei Lin, James Lincoff, Stefan Linder, Karin Lindkvist, Grace Lindsay, Keith Lindsey, Anna Lindstrand, Linda Lindström, Kun Ling, Shuo-Chien Ling, James Linton, Yih-Cherng Liou, Jan Lipfert, Dina Lipkind, Guy G. Lippens, Cat Lippi, Daniel Lipus, Sabrina Lisboa, Mike Litzow, Bao Liu, Bin Liu, Bo Liu

Chang Liu, Changning Liu, Chaohong Liu, Clarissa Liu, Dongfang Liu, Fei Liu, Feng Liu, Fenyong Liu, Guanghui Liu, Hong-Xiang Liu, Hou-cheng Liu, Jian Liu, Jianquan Liu, Jinxin Liu, Jun Liu, Kangze Liu, Kathy Fange Liu, Liang Liu, Lin Liu, Meilian Liu, Qiang Liu, Qing Liu, Quan-Xing Liu, Qun Liu, Renjing Liu, Rujuan Liu, Sanzhen Liu, Shanlin Liu, Sheng Liu, Shiyong Liu, Tzu-Ming Liu, Xiaojing Liu, Ya-Hong Liu, Ya-Wen Liu, Yang Liu, Yen-Nien Liu, Yuan Liu, Yuxi Liu, Zhanju Liu, Zhi-Ping Liu, Zhibo Liu, Zhijie Liu, Upekha Liyanage, Catherine Llanes, Roland Lloubes, Joyce Lloyd, Chien-Jung Lo, Jason W. Locasale, Dan Loeb, Bart Loeys, Markus Löffler, Kristopher A. Lofgren, Elsa Logarinho, Delphine Logeart-Avramoglou, Diomedes E. Logothetis, Jacelyn (Mei San) Loh, Josef Loidl, Claus J. Loland, Zane Lombard, Giovanna Lombardi, Fanxin Long, Hannah Long, Heather Long, Edoardo Longarini, Maria Paola Longhi, Tapio Lönnberg, Céline Loot, Gary Lopaschuk, Allison Lopatkin, Coeli Lopes, Jose Maria Lopez Ayala, Diana Lopez-Barosso, David López-Escardó, Purificación López-García, Alizee Lopez-Persem, Ruben Lopez-Vales, Andrew Lopez, Víctor Lorenz-Fonfria, Aurelio Lorico, Cecile Lorrain, Aba Losi, Shaun Lott, Swarnali Louha, Antoine Louveau, John C. Love, E Joel Loveridge, Leanne M. Low, Jan Löwe, Winsor Lowe, Noel F. Lowndes, Chao Lu, Jia-Hong Lu, Jin-Jian Lu, Maixin Lu, Mingyang Lu, Qiongshi Lu, Zhong Lin Lu, Francesca Luca, Christopher Lucas, Artee Luchman

Shirley Luckhart, Marian Ludgate, William Ludington, Vivian W. Lui, Angie Luis, Soeren Lukassen, Maria M Lukina, Valeria Lulla, Peter A. Lund, Iben Lundgaard, Claudia Lunghi, Marine Lunven, Arong Luo, Hong Luo, Yun L. Luo, Christopher Lupfer, Patrick Lusk, Joe Lutkenhaus, Michael Lutz, Sarah Lutz, Jiale LV, Gareth Lycett, David Lydon-Staley, Frank Lyko, Michael D. Lynch, Angeline M. Lyon, Leslie Lyons, Lisa C. Lyons, Michael Lyons, Dmitry Lyumkis, Donald M. Kuhn, Pedro Manoel M. Moraes-Vieira, Mohsin M. Naqvi, Hairong Ma, Jian Ma, Lijia Ma, Shuyi Ma, Yutao Ma, Z Ma, Zhenyi Ma, Jacqueline R. Maasch, Karen Maass, Juliano Machado, Carolyn Machamer, Erika Machtinger, Charles Mackay, Joel P. Mackay, Trudy Mackay, Iain J. MacLeod, Narayanan Madaboosi, Paolo Madeddu, Marvin Maechler, Yutaka Maeda, Steven Maere, Sebastian Maerkl, Dirk Maes, Enrico Magnani, Magnus K. Magnusson, Chidambareswaren Mahadevan, Bidesh Mahata, Joseph Maher, Michael Maher, Ari Pekka Mähönen, J M. Mahoney, Ana Teresa Maia, Helder Maiato, Jessica L. Maiers, Richard E. Mains, Tushar K. Maiti, Sourav Maity, Michelle Maiworm, Samir K. Maji, Angel C.Y. Mak, Pavel Makarevich, Eugene Makeyev, Thulani P. Makhalanyane, Anna R. Malacrida, Hannah Malcolm, Mirjana Maletić-Savatić, Anna Maria Malfitano, Rayaz Malik, Bernard J. Malissen, Erich P. Malkemper, Safia Malki, Maneesha Mall, Srinivas Malladi, Nicolas Mallet, Sandeep Mallipattu, Bettina Malnic, Susan Maloney, Tadanori Mammoto, Cristina Mammucari, Dileep Mampallil, Erika Mancini, Nicholas Mancuso, Chitra Mandyam, Giovanni Manfredi, Pierre H Mangin, Sridhar Mani, Ani W. Manichaikul

Richard Mann, Madhu Manna, Hanna Mannell, Mauro Manno, Nicholas Manoukis, Benedicte Manoury, Michael D Manson, Nahal Mansouri, Sheref S. Mansy, Jennifer Manuzak, Angelo Mao, Youdong Mao, Adam Mar, Costas Maranas, Ambroise Marcais, Matteo Marcantonio, Asaf Marco, William Margolin, Abelardo Margolles, Carla Margulies, David H. Margulies, Jan Marienhagen, Daniele Marinazzo, Giovanni Marini, Ilaria Marinoni, David B. Mark Welch, Brian L Mark, Fritz Markwardt, Sylvie Marleau, Eirini Marouli, Drew Marquardt, Nicole Marquardt, Rui Filipe Marques Lopes, Fernando Marqués-García, Chantal Marquez, Rogier B. Mars, Joseph A. Marsh, Sarah Marshall-Pescini, C Marshall, Lee Marshall, Jaime Martin-Benito, Marisa L. Martin-Fernandez, Vincent J. Martin, Marcel Martínez Porchas, Rosa María Martínez-Espinosa, Alino Martinez-Marcos, Jose Martinez-Navio, Alessandra Martini, Sabata Martino, Daniele Martins, Gabriel G. Martins, Gustavo Martins, Anna Martner, Sandra R. Maruyama, Atsushi Masamune, Martin Mascher, Stefania Masci, Cecilia Mascolo, Robin L. Maser, Rosalinde Masereeuw, Jon Massey, Glenn Masson, Ruturaj Masvekar, Dorota Maszczak-Seneczko, Marjolaine Matabos, David Matallanas, Sadis Matalon, Ponpan Matangkasombut, Marion Mathelié-Guinlet, Sijo Mathew, Patrick Mathieu, Anukriti Mathur, Ramkumar Mathur, Ajay S. Mathuru, Ivan Matic, Kenta Matsuda, Tomoki Matsuda, Tracie Matsumoto, Hiroaki Matsunami, Toshiya Matsushima, Tomoaki Matsuura, Ben Matthews, Brya Matthews, David A. Matthews, Steffi Matthyssen, Daumantas Matulis, Daniel Matute, Oriane Mauger, Andrew Maurer, Arne May, Harold May, Sylvio May, Victor May, Klaus F. Mayer, Alejandro E. Mayorca-Guiliani, Claire Mazahery, Alison McAfee, Samuel McBrayer, Luke McCaffrey, J Scott McCain

Jordan G. McCall, Alex J McCarthy, James Joseph McCarty, Owen McCarty, David McCauley, Tim R. McClanahan, Colleen A. McClung, Ryan McClure, Katherine McCormack, John K. McCormick, David J. McCulley, Michael McDonald, Robert McDonald, Caitlin McDonough-Goldstein, Ian McDonough, Jennifer McDonough, Diane McDougald, Iain McEwan, Margaret McFall-Ngai, James McFarland, Vincent McGinty, Jacqui A. McGovern, Sheena McGowan, Leeanne McGurk, Thomas McHugh, Andrew McIntosh, Duncan McKenzie, Linda McLoon, Aoife McLysaght, Mark McMullan, Simon McMullan, Alan McNally, Maria E. McNamara, Joseph B. McPhee, Allan McRae, Julien Meaud, Karl Mechtler, Ohad Medalia, Maria Medalla, Pedro A. Mediano, Richard R. Meehan, Rene-Marc Mege, Akira Meguro, Carolina Mehaffy, Shahid Mehmood, Jie Mei, Florian Meier, Matthias Meier, Sigolene Meilhac, Sigolène M. Meilhac, Joyce C. Meiring, Giuseppe Melacini, Anat Melamed, Emmanuel Mellet, Maria Luiza S. Mello, Stephano Mello, Roman A. Melnyk, Fani Memi, **Daniel Mende [4]**, Jerome Menet, Jia Meng, Daniela Mennerich, Benedetta Mennucci, Georges Mer, Ismail M. Meraz, Gildas Merceron, George Merces, Enrique Mesri, Ilhem Messaoudi, Joris Messens, Karl Messlinger, Julia Metzger, Anne Meyer, Rikke Louise Meyer, David Meyre, Msizi Mhlongo, Mike Michaelides, Kelly Michaelis, Signe R. Michaelsen, Giorgia Michelini, Michel-Edwar Mickael, Ainhoa Mielgo, Claudia T. Mierke, Joel Miesfeld, Constantinos M. Mikelis, Alexander S. Mikheyev, Martin Mikl, Dusanka Milenkovic, Lorin Milescu, George Miller, Jeremy A. Miller, Laura Miller, Laurence J. Miller, Miles A. Miller, Jean K Millet, Carol Milligan, Julia Milne, Justin Milner, Pohl Milón, Miltyk Miltyk

Byoung-Kyong Min, Wei Min, Rinki Minakshi, Yusuke Minato, Akira Mine, Gabriel Antonio S. Minero, Yixuan Ming, Bui Q. Minh, John D. Minna, Samuel Minot, Lisa Miorin, Nuno Mira, Cindy K Miranti, Yekaterina Miroshnikova, Peter Mirtschink, Razmik Mirzayans, Santosh K. Mishra, Satish Mishra, Michele Mishto, Bratislav Misic, Hari S. Misra, Prashant Misra, Sanjay Misra, Pawel Misztal, Thomas Mitchell-Olds, Caroline Mitchell, Daniel Mitchell, Kara Mitchell, Simon Mitrovic, Chitvan Mittal, Akio Miyamoto, David Miyamoto, Kei Miyamoto, Makoto Miyara, Osamu Miyashita, Kohei Miyazono, Kiyofumi Miyoshi, Petra Mlcochova, Qianxing Mo, Ken Moberg, **Mehdi Mobli [4]**, Kazufumi Mochizuki, Thomas Mock, Leonhard Moeckl, Emad Moeendarbary, Bruno Moerschbacher, Smita Mohanty, Ian Mohr, Wendy Mok, George-Lucian Moldovan, Miriam Molina Arcas, Mark Moline, Mojtaba Mollaei, Nardus Mollentze, Julia Molnar, Dmitry Molodenskiy, José Carlos Mombach, Amal Mondal, Stephen J. Mondo, Alexander A. Mongin, Carrie Mongle, Viviana Monje-Galvan, Nicole Mons, Alon Monsonego, Xavier Montagutelli, Antonio Monteiro, Hugo P. Monteiro, Luana C. Monteiro, Marta Montes, John Montgomery, Francesco Montinaro, Kristi Montooth, Kevin Moon, Emily R. Moore, Mark Moore, Peter B. Moore, Torben Moos, Héctor M. Mora-Montes, Freddy Mora, Maximilian Mora, Joanna Moraczewska, Trevor F. Moraes, Livia Morais, Tiago Morais, Tomas Moravec, Kelley Moremen, Herman Moreno, Solange Moréra, Rosa Maria Moresco, Nicholas Morffy, Duncan Morgan, Michael Morgan, William Morgan, Diego Morgavi, Munemasa Mori, Eiichi Morii, Antonin Morillon, Jumpei Morimoto, Shai Morin, Masahiro Morishita

Naoki Morita, Ryuji Morizane, Ken-ichirou Morohashi, Toshiro Moroishi, Anna Moroni, Nadya Morozova, Erin Morrison, Meghan Morrissey, Michael Morrow, Marco Morselli, Timothy J. Mosca, Anne Moscona, Gregory Moseley, Yevgeny Moskovitz, Cynthia Moss, Karen Mossman, Maria M. Mota, Rajender K Motiani, Joana Mourao, Henrik Mouritsen, Yvonne Mowery, Masoud Mozafari, Andrea Mozzarelli, Natalie Mrachacz-Kersting, Dezhi Mu, Ping Mu, Ulrich Muehlenhoff, Scott N. Mueller, Stephen P. Muench, Carina F. Mugal, Joseph Mugleston, Samwel Muiruri, Yukio Mukai, Srabani Mukherjee, Partha Mukhopadhyay, Karolina Mukhtar, Shahid Mukhtar, Carla Mulas, Mark Muldoon, Michaela Muller-McNicoll, Gernot Müller-Putz, Eli Muller, Florian L. Muller, Johannes Müller, Lyle Muller, Rolf Müller, Philip M. Mullineaux, Alex Mullins, Erin Mulvihill, Jeanette A. Mumford, Christian Münch, Jan Münch, Jorge Munera, Lucile Muneret, Altar M. Munis, Patrick Munk, Lance Munn, Carmen Muñoz-Ballester, Rosario A Muñoz-Clares, Sean Munro, Mathias Munschauer, Mary Munson, Aura Muntasell, Santoshi Muppala, Junko Murai, Samuel Murail, Brenda Murdoch, Minae Mure, Pablo Muriel, Kristin Murphy, Patrick Murphy, Timothy H. Murphy, John Murray, M.R.N. Murthy, Mala Murthy, Richard Muscat, Catherine Musselman, Marko Mutanen, Serge Muyldermans, Alexander Myburg, Shreesh Mysore, Karthikeyan Mythreye, Kyungjae Myung, Nagalakshmi Nadiminty, Rachael Naegele, Sahana Nagabhushan Kalburgi, Takeharu Nagai, V Nagaraja, Ravinder Nagpal, Laszlo G. Nagy, Saidas Nair, Smita Nair, Dean J. Naisbitt, Raid Naji, Yusaku Nakabeppu, Hidewaki Nakagawa, Ichiro Nakagawa, Shinichi Nakagawa, Yasukazu Nakahata, Yuki Nakamura

Akihiko Nakano, Toshiyuki Nakata, Karina Nakayama, Harikrishna Nakshatri, Jin-Wu Nam, Yunsun Nam, Dhanya K Nambiar, Vilis Nams, Xiaolin Nan, Tulika Nandi, Sahin Naqvi, Akshay Narkar, Carla Nasca, Pierre Nassoy, Samuel Nastase, Paolo Natale, Kedar Natarajan, Matthias Nau, **Richard Naud [4]**, Surya Nauli, Shaghayegh Navabpour, Joaquín Navajas, Fernando Navarro-Garcia, Vikas Navratna, Stephen Nayfach, Richard Naylor, Anand Nayyar, Matthew Neave, Stephan Nebe, Andreas Nebenführ, Leonard Neckers, Leonard M. Neckers, Frauke Nees, Albrecht Neesse, Matthew Nelsen, David R. Nelson, David Nemazee, Ujjwal Neogi, Paola Neri, Peter Neri, Tracy Nero, Wladimir Neto, Inga Neumann, Ines Neundorf, Irene Neuner, Helia Neves, Kelly Newell, Aaron M. Newman, Alexandra AC Newman, Phillip Newton, Esther Ng, Ho-Leung Ng, Tin Nguyen, Tuyen Nguyen, Heyu Ni, Raffaele Nicastro, Chris Nice, Colin G. Nichols, Robert W. Nickells, Alain G. Nicolas, Chiara Nicolazzo, Yvain Nicolet, Qing Nie, Caroline Nieberding, Ashley Nielsen, Rienk Nieuwland, Sergey Nikolaev, Nikolas Nikolaidis, Marko Z. Nikolic, Zoran Nikoloski, Matthew Nilles, Andre Nilsen, Eric Nilsson, Dominik Niopek, Yuji Nishikawa, Riko Nishimura, Ryuichi Nishinakamura, Koki Nishitsuji, Atsuya Nishiyama, Hitoshi Niwa, Alex J. Noble, Christopher Nobles, Tatsuya Nobori, Jean-Paul Noel, Ana Nogal, Yasuki Noguchi, Juan Nogueira, Yvonne Nolan, Wataru Nomura, Rashed Noor, Daan Noordermeer, Lorena Norambuena, Parisa Norouzitallab, Russell A. Norris, **Georg Northoff [4]**, Reuben Nowell, Victor Nozais, Valentine O. Ntui, Francesco Nugnes, Bramasta Nugraha

Francis Nunes, Zoltan Nusser, Ruth Nussinov, Patricia Nuttall, Dale R. Nyholt, Edward P. O’Brien, John O’Brien, Reuben D. O’Dea, Eoin J. O’Gorman, Andrias O’Reilly, Christian O’Reilly, Richard O’Reilly, Jeremy O’Sullivan, Ronan O’Toole, Clint Oakley, Fumiaki Obata, Darren Obbard, Felix Oberhauser, Georg Oberhofer, Nadine Obier, Miroslav Obornik, David O. Obura, Raul Ochoa-Hueso, Rodrigo Ochoa, Michael Ochsenkuehn, Brian Odegaard, Thomas G. Oertner, Vibe Oestergaard, Bergithe E. Oftedal, Hideaki Ogata, Atsuko Ogino, Yong-Seok Oh, Kazutaka Ohi, Yukari Ohki, Joyce Ohm, Hiroshi Ohno, Shigetoshi Oiki, Nikos Oikonomou, Ui Okada, Hirohiko Okamura, Katsutomo Okamura, Yasushi Okamura, Freya Olafsdottir, Emily Olafson, Aida Oliván-Viguera, Cecilia Oliver, Rebekah Oliver, Richard P. Oliver, Angela Oliverio, Coux Olivier, Michael S. Olsen, Elizabeth Olson, Kyla D. Omilusik, Linda Oña, Jose Onata-Garzon, Derrick Ong, Edison Ong, Ju Lynn Ong, Weg Ongkeko, Akinyemi Oni-Orisan, Itay Onn, Daisuke Ono, Noriaki Ono, Shoichiro Ono, Yaw-Shin Ooi, Hiroki Oota, Michael Opata, Patricia L. Opresko, Enrico Opri, Richard Oram, Alexander Orekhov, Elena V. Orlova, William D. Orsi, Caitlin Orsini, Jesus Osada, Thanaphum Osathanon, Tsuyoshi Osawa, Yohei Oseki, Richard Ostfeld, Julie Ostrander, Lev Ostrer, Serge Ostrovidov, Jessica Oswald, Maria Oszvald, Gard W. Otis, Timo Otonkoski, Jente Ottenburghs, Christian Ottmann, Michael Otto, Oliver Otto, Daniel Otzen, Le Ou-Yang, Madeleine Oudin, Michael Overduin, Catherine E. Ovitt, David D. Owen, Shawn C. Owen, Michelle Oyen, Suat Özbek, Engin Ozcivici

Hakime Öztürk, Andrzej Pacak, Emilie Pacary, Douglas Pace, Valentina Pacella, Martin Pacesa, Timothy P. Padera, Oliver Padget, Benjamin Padilla, Vasantha Padmanabhan, Pablo M. Paez, Annie Page-Karjian, Malcolm Page, Richard Page, Stefano Pagliara, Oliver Pain, Kevin Painter, Erola Pairo-Castineira, Sandra Paiva, Giulia Palermo, Prasit Palittapongarnpim, Carla Pallavicini, Komaraiah Palle, Wilhelm Palm, Clarisse Palma-Silva, Magnus Palmblad, Rupert Palme, Andrew Palmer, Stefano Palminteri, Francesca Palombo, Yannis Paloyelis, Bernhard O. Palsson, Stephen S. Palumbi, Satu Palva, Chongle Pan, Dongli Pan, Feng Pan, Guoyu Pan, Jiahui Pan, Lurong Pan, Xuefeng Pan, Eleftheria Maria Panagiotou, Abhimanu Pandey, Avinash C. Pandey, Prajita Pandey, Veethika Pandey, Lucas Paneiras-e-silva, Stephanie A. Pangas, Pierpaolo Pani, Stanislava Pankratova, Wimonrat Panpetch, Antonios Pantazis, Alkmini Papadopoulou, Gabor Papai, Rohit V. Pappu, Dominic Paquin-Proulx, Emmanuel Paradis, Isabel Pardo, Luba Pardo, Luis A. Pardo, Caroline Parins-Fukuchi, Bo-yong Park, Daehun Park, Eun Jeong Park, Eunyong Park, Hyun Ho Park, Jeehye Park, Joon Seok Park, Young-Kyoung Park, Joel Parker, Kevin Parsons, Matthew Parsons, Sarah Partan, Raghavendran Partha, Aravindakshan Parthasarathy, Mark R. Parthun, Anthony W Partridge, Kislay Parvatiyar, Josef Parvizi, Maria Pastina, Ievgenia Pastushenko, Ardem Patapoutian, Kash Patel, Smita Patel, Simone Patergnani, Kavitha Pathakoti, Chris Patrick, Paola Patrignani, Sitakanta Pattanaik, Matthew Paul, Joao A. Paulo, Ian T. Paulsen, James C. Paulson, Robert Paulson, Kajsa M Paulsson, Patrick Pausch, Francesco Pavone, Paulina Pawlica, Teresa E. Pawlowska, Claire Peart

Francois J. Peaudecerf, Diana Peckys, Chad V Pecot, Claire Pecqueur, Shyamal Peddada, Brent Pedersen, Roger Pedersen, Thais Pedrete, Nicolas Pégard, Michael Peitz, Murilo Peixoto, Serena Pellegatta, Laura Pellegrini, Sabine Pellett, Kevin Pelphrey, Eduardo J. Pena, Brandt D. Pence, Bo Peng, Yao-Jin Peng, Zhihai Peng, Peter Penson, Francesca Pentimalli, Matjaž Perc, Avi Peretz, Mileidys Perez-Alea, Rolando Perez-Lorenzo, Alan Perez-Rathke, Alejandro Perez, Camilo Perez, Paulino Pérez, Muthu Periasamy, Cyril Pernet, Christopher Perry, Rachel J. Perry, Jenny Persson, Mihaela Pertea, Angel V. Peterchev, David Peterman, James Petitte, Petko Petkov, Ezequiel Petrillo, Pavel A. Petukhov, Donald W. Pfaff, Suzanne R. Pfeffer, Brett Pflugrath, Fortunate Phaka, Thanh K. Phan, Otto Phanstiel, Christopher Philipson, Julie Phillippi, Alon Philosof, Swastik Phulera, Xinchun Pi, Giulia Piaggio, Anne Piantadosi, Martin Picard, Stefano Piccolo, Sebastien Pichoff, Elah Pick, Anthony Pickering, Gary Pielak, Rian Pierneef, Paolo Pierobon, Darren Pietersen, Marie Pigeyre, Astikainen Piia, Jordy Pijl, Pim Pijnappel, Prakash Pillai, Ramesh Pillai, Nicolas Pilon, Dan Pina Fuentes, David Pincus, Jarone Pinhassi, Lorenzo Pini, Lea Pinon, Dorothea Pinotsi, Stefan Pinter, Paola Piomboni, Alberto Piperno, Nicola Pirastu, Andreas Pircher, Rosario M Piro, Liise-Anne Pirofski, Antonio Pisani, Doris Pischedda, Geoffrey Pitt, Michael Pittman, Jason Pitts, Eric Piver, Vittorio Pizzella, Dimitris Placantonakis, Antoni Planas, Mireya Plass, Michelina Plateroti, Maksim V. Plikus, Jonathan Ploski, Larissa M. Podust, Erik H Poelman, Alessandro Poggi

Igor Pogribny, Jia-Hou Poh, Mathilde M. Pohin, Sebastian &. Pohl, Yury S. Polikanov, Renato Polimanti, Jonathan Polimeni, Thad Polk, Tim Pollex, P David Polly, Luca Polonio, Frederic Poly, Steve Polyak, Constantin Polychronakos, Jean-François Pombert, Matthew Pomrenze, Evgeni Ponimaskin, Luigi Ponti, Tzvetan Popov, Cristina Porcheri, Marcin Poreba, Michael Portelli, James Porter, Gereon Poschmann, Karen L Posey, Arnaud Poterszman, Marie-Claude Potier, Celio Pouponnot, Jean-Luc Poyet, Gabriele Pradel, Amynah Pradhan, Laurent Pradier, Jerome Prado, Steven Prager, Shaurya Prakash, Amit Prasad, Rajendra Prasad, Sebastian Preissl, Diego M. Presman, Robert Prevedel, Avi Priel, Daniel R Principe, Anne Pringle, Ian Prior, William Probert, Richard Proctor, Emmanuel Procyk, Heike Pröhl, Jeremy Prokop, Ludmila Prokunina-Olsson, Mélanie Pruvost, Holger Puchta, Christopher W Pugh, Raffaelo Pugliese, Marta Puig, Erdem Pulcu, Enkhsaikhan Purevjav, Sunil Puria, Rahul Purohit, Michael D. Purugganan, Roland Pusch, Gerhard P Püschel, Smruti Pushalkar, Xiaoquan Qi, Yiping Qi, Li Qian, Xuyu Qian, Zhaohui Qian, Liang Qiao, Yi Qiao, Enya Qing, Jiangxiao Qiu, Qiang Qiu, Yin-Long Y. Qiu, Zhihua Qiu, Que Qiudeng, Linbing Qu, Yang Qu, Loredana Quadro, Robert B. Quast, Corbin Quick, Kyle P. Quinn, Tobias Raabbe, Ton J. Rabelink, Roy Rabinowitz, Ralf Rabus, Chloe Rackham, Noura Raddadi, Annalisa Radeghieri, Christoph Rademacher, Ute Radespiel, Zoran Radić, Aleksandra Radosavljevic, Sylvain Raffaele, Md Mizanur Rahman, Mizanur Rahman, Muhammad Rai, Joe Rainger, Roberto T. Raittz, Hamed Rajabi

Srinivasan Ramachandran, Hazem Ramadan, Vetrivelan Ramalingam, Priya Raman, Saravana Ramasamy, Robert Rambo, C.H. Ramesh, Pranela Rameshwar, Mirana Ramialison, Jan Marino Ramirez, Kelly Ramirez, Elena Rampanelli, Simone Rampelli, Rona Ramsay, Armelle Rancillac, Padmini Rangamani, Christopher Ranger, Erinn B. Rankin, Ponugoti Vasantha Rao, Rajini Rao, Xiancai Rao, Jayne Raper, Vera Lúcia Raposo, Leda Raptis, Mladen-Roko Rasin, Torben S. Rasmussen, Jonathan Rast, Mojgan Rastegar, G. Ratcliffe, Rinki Ratnapriya, Christopher Raub, Terje Raudsepp, Michael J. Rauh, Catherine Ravel, Shoshana Ravid, Shilpa Ray, Sumanta Ray, James Raymond, Pamela Raymond, Shan E Ahmed Raza, Suyong Re, Paul Rea, Clare Rebbeck, Ignacio Rebollo, Manuele Rebsamen, Meital Reches, Juan A. Recio, Marianne Reddan, Hemachandra Reddy, Sekhar P. Reddy, Geraint Rees, Helen C. Rees, Paul Rees, Dora Reglodi, Finn Rehling, Hansotto Reiber, Shannon Reilly, Jüri Reimand, Francesco Reina, Judith Reindl, John Reinfelder, Kurt Reinhart, Lukas Reiter, Philippe Remigi, Guangwen Ren, Tianying Ren, Xiaojun Ren, Fabian Rentzsch, Jonathan Repple, Elizabeth Rettedal, Scott T. Retterer, Miriam Reverter, Thomas Rexer, Natalie Reznikov, Ki-Jong Rhee, Kunsoo Rhee, Timothy Rhoads, Jack Rhodes, Justin Rhodes, Domenico Ribatti, Andrew P Rice, Suman Rice, William J. Rice, Jeremy N. Rich, Peter Richard, Blake Richards, Stephen Richards, Goetz Richter, Peleg Rider, Diana Riebold, Christiane Riedel, Bernd Rieger, Rodrigo Riera, Olaf Riess, Ricardo Soto Rifo, Lionel Rigottier-gois, Jason Rihel, James K. Rilling, Tyler Hyungtaek Rim, Johanna Rimmele

Paolo Rinaudo, Malene Ringkjøbing Jensen, Maria Giovanna Riparbelli, Pablo Ripollés, Olivia Rissland, Ken Ritchie, Juan Pablo Rivero Vaccari, Aaron Robart, Stefanie Robel, Francois Robert, Anna I. Roberts, James Roberts, Leah Roberts, Todd Roberts, Charles Robin, Peter Robinson, Pierre Roblin, Paul Robustelli, Joana Rocha, Jean-Christophe Rochet, Pedro Roda-Navarro, Rosario Rodicio, Marina V. Rodnina, Ana Rodrigues, Juan-Carlos Rodríguez-Manzaneque, Aldo A. Rodríguez-Menchaca, Sandra Rodríguez-Perales, Cesar Rodriguez-Saona, Francisco Rodriguez-Valera, Gabriela Rodriguez, Karl Rodriguez, Connor Rogerson, Lars Rogge, Ali Roghanian, Hyun Roh, Tibor Rohacs, Gustavo Rohenkohl, Till A. Röhn, Nicolas Rohner, David Lucas Rohr, Laila Roisman, Sofia Roitman, Charlotte Rolny, Danilo Roman-Campos, Roman Romanov, Adriana Romero-olivares, Vittoria Roncalli, Yang Rong, Yueguang Rong, Zhili Rong, Igor B Roninson, Fredrik Ronquist, Nicola Rooney, Diana Roopchand, Jatin Roper, Marcus Roper, Nuria Ros, Fernando Rosas, **Richard E. Rosch [4]**, Stefan Rose-John, Stacy Rosenbaum, Kobi Rosenblum, Soren Rosendahl, Samantha Rosenthal, Andrew J. Ross, Jason Ross, Robert Ross, Carine Rossé, Holly Rossiter, Wilfried Rossoll, Rossella Rota, Robert A Roth, Alissa Rothchild, Markus Rothermel, Kenneth Rothschild, Veerle Rottiers, Lisa Röttjers, Brice Rotureau, Marion Rouault, Kyle Rouen, Virginie Rougeron, Vassilis Roukos, Jennifer Round, Alain Roussel, Kyle J. Roux, Annette Rowe, Owen Rowland, Jordan Rowley, Dipanjan Roy, Dominic Roy, Polly Roy, Jessica Royer, Wilfried Rozhon, Sara Ruane, Dustin R. Rubenstein, Halina Rubinsztein-Dunlop, Peter Rudebeck, Daniel Rudic, Joan Rué Queralt, David S. Rueda

Markus A. Ruegg, Marc-David Ruepp, Francesco S. Ruggeri, Gennaro Ruggiero, Laura Ruiz-Aceituno, Andrés Ruiz-Linares, Marcel Ruiz-Mejias, Edward A. Ruiz-Narváez, Fernando Ruiz-Pérez, Caroline A. Runyan, Stephen J. Russell, Alessandro Russo, Annapina Russo, Christopher Russo, Thomas Russo, Arjun Rustagi, Kate Ruth, Julie Rutten, Emma Rybalka, Bernard Ryffel, Bernhard Ryffel, Sephira Ryman, William Ryu, Gaurisankar Sa, Lehti Saag, Federico Sabbadin, Morteza Saber, Susanna Sabin, Zakee Sabree, David B. Sacks, David D. Sacks, Aiko Sada, Sepideh Sadaghiani, Sadra Sadeh, Perot Saelao, Ignacio Saez, Juan Carlos Saez, Mohammad Reza Safaei, Saveez Safarrian, Daad Saffarini, Paul Saftig, Ronit Sagi-Eisenberg, Gaurav Sahay, Mehmet Sahin, Helen R. Saibil, Supreet Saini, Yoshifumo Saisho, Shoko Sakai, Saori Sakaue, Lori C. Sakoda, Carlo Sala, Roberta Sala, Asaf Salamov, Ali Salanti, Lucas A. Salas, Mary Salcedo, Shahram Salek-Ardakani, Emilio Salinas, Ahmed Salman, Viljami Salmela, Jordi Salmona, Robert Salmond, Davinia Salvachua, Shayla Salzman, Hassan Samee, Alapakkam Sampath, Harini Sampath, Timothy Sampson, Robert Samstein, Mehmet K. Samur, Alejandra San Martin, Karissa Y. Sanbonmatsu, M Virginia Sanchez-Puerta, Carlos Óscar Sánchez-Sorzano, Clara Sanchez, Diego Sanchez, Patricia Sancho, Johan K. Sandberg, Charles R. Sanders, Jon Sanders, Yana Sandlers, Rebecca Sandlin, Ioana Sandu, Joshua Sanes, Dominique Sanglard, Rajesh K. Sani, Andrea J Sant, Sabato Santaniello, Arvind Santhanakrishnan, Tasha Santiago-Rodriguez, Stephen W. Santoro, Teresa Santos-Mendoza, Dulce Santos, Mafalda Santos, Margarida Saraiva, Pinaki Sarder, Suparna Sarkar-Banerjee, Hugo Sarmento, Hiiroki Sasaguri, Ken Sasaki

Takehiro Sasaki, Tsutomu Sasaki, Takayuki Sassa, Anuja Sathe, Akiko K Satoh, Abhay Satoskar, Kyle Satterstrom, Rita Sattler, Bruno Sauce, Philippa TK Sauders, Jaclyn Saunders, Dominic Sauvageau, Jimmy Saw, Kazunobu Sawamoto, Ritwick Sawarkar, Deepak Saxena, Jesse Sayles, Diego Sbardella, Giuliano Scarcelli, Eliana Scemes, Stephen Schaffer, Isabelle J. Schalk, Jack A. Schalken, Anett Schallmey, Christoph Schaniel, Daniel Schardosim Calovi, Michael E Scharf, Constance Scharff, Stefan Schattgen, Ferenc A. Scheeren, Alexander Scheffold, Christoph Scheiermann, Dustin Scheinost, Sebastian Schenk, Katja Schenke-Layland, Katharina Scherer, Stephen W. Scherer, Sjors H. Scheres, István Scheuring, Torsten M. Scheyer, Andreas Schiermeyer, Helmut Schiessel, Philipp Schiffer, Niels O. Schiller, Joshua P. Schimel, Johannes Schittny, Martin Schlegel, Dietmar Schlosser, Tilman Schlothauer, Benjamin Schmid, Michael Schmid, Amand F. Schmidt, Carla Schmidt, Liane Schmidt, Marius Schmidt, Thomas Schmidt, Miriam Schmidts, Gerold Schmitt-Ulms, Clemens A. Schmitt, Oswald J. Schmitz, Monika Schmoll, Robert Schnabel, Dirk Schnappinger, Rene Schneider, Till Schneider, Dina Schneidman, David Schnyer, Kilian Schober, Ethan A. Scholar, Carsten C. Scholz, Hanne Scholz, Ernst Schonbrunn, Erwin M. Schoof, Joseph M. Schrader, Martina Schröder, Elena Schroeter, Werner Schroth, Gerhard Schuetz, Shimon Schuldiner, Ulrich Schüller, Stefan Schulte-Merker, Carsten Schulte, Benjamin Schulz, Thomas F Schulz, Ben Schumann, Florian K. Schur, Haiko Schurz, Gerhard J. Schütz, Margot Schwalbe, Martin A. Schwartz, Russell S. Schwartz, Erich M. Schwarz, Martin Schwemmle, Alison Scott, Charlotte Scott, Daniel J. Scott, Emma Scott, Marco Scotti, Alexander Sczyrba, Peter Sebo

Julie Secombe, Will Sedley, Esen Sefik, Loes I. Segerink, Garima Sehgal, Max Seibold, Tal Seidel Malkinson, Paul Seidler, Mark Seielstad, Aaron Seitz, Sellappan Selvaraju, Jayson Semmens, Ranjan Sen, Nimai Senapati, Donna Senger, Amitava Sengupta, Inna Senina, Bo Ri Seo, Jihye Seong, Kristina Sepčić, Jorge Sepulcre, Mattia Serra, Ángel Serrano-Aroca, Apandra Sertil, Joseph Servadio, Irina Serysheva, Guillaume Sescousse, Evodia Setati, Ole-Morten Seternes, Carmine Settembre, David Seung, Lisa Sevenich, Mathew Seymour, Brett Seymoure, Haitham (Ahmed) Shaban, David Shackelford, Aaron B. Shafer, Bridget Shafit-Zagardo, Yavin Shaham, Migun Shakya, Shu-ou Shan, Feng Shao, Huilin Shao, Mingfu Shao, Yongqi Shao, Zhi-Ming Shao, Ankur Sharma, Anup Sharma, Dipali Sharma, Pushkar Sharma, Tapan Sharma, Andrew Sharott, Joshua S. Sharp, Koty Sharp, Tatyana O. Sharpee, Prasad Shastri, Igor Shats, Yosef Y. Shaul, Jordan Shavit, Colin Shea, Nazleen Sheikh, Skand Shekhar, Adam Shellard, Jason M. Sheltzer, Jian-Ren Shen, Jie Shen, Qichen Shen, Qingtang Shen, Xiaotao Shen, Xiling Shen, Xueyi Shen, Yin Shen, Yudao Shen, Douglas Shepherd, Elion Sherman, Emma Sherratt, Eric Sheu, Guo-Ping Shi, Jian-Yu Shi, Kilin Shi, Xiaorui Shi, Zheng-Li Shi, Shigeru Shibata, Brian Shiels, Katsunori Shigehara, Andy Shih, David J. Shih, Hong-Yan Shih, Joon Shim, Sang-Hee Shim, Mikio Shimada, Jae-Won Shin, Yuki Shindo, **James M. Shine [4]**, Norifumi Shioda, Kari Ann Shirey, Elena Shklovskaya, Andrey N. Shkoporov, Sarah Shomstein, Danielle Shore

Francesca L. Short, James Shorter, John Shorter, Harel Shouval, Nick Shrine, George Shubeita, Wenqing Shui, Girish C. Shukla, Sanjeev Shukla, Ke Si, Xingfeng Si, John-William Sidhom, Bettina Siebers, Dirk Sieger, Stefan Siemann, Alejandra Sierra, Nourridine Siewe, Alice Sijts, Anna Sikora, Katarzyna Sikora, Arita Silapetere, Eva Siles, Alba Silipo, Joan J. Silk, David Sillam-Dussès, Alcino Silva, João Pedro Silva, David L. Silver, Ludovico Silvestri, Daniele Silvestro, Prashanta Silwal, Constantine A. Simintiras, Blake A. Simmons, Marcos Simoes-Costa, Zilá Simões, Carlos Simon, Meinhard Simon, Thomas Simon, Douglas Simonetto, Michel Simonneau, Matthew Simpson, Rebekah Sinclair, Oleg A. Sineshchekov, Amar Singh, Anugrah Singh, Gatikrushna Singh, Harpreet Singh, Praveen K. Singh, Ravail Singh, Ravindra Pal Singh, Ritambhara Singh, Ruchira Singh, Abhishek Singharoy, Simarjit Singhrao, Abhishek Sinha, Debajyoti Sinha, Sanjay Sinha, Sanjiv Sinha, Musalula Sinkala, Flore Sinturel, Konstantinos Siontis, Anton Sirota, Nicholas J. Sisco, Jacobo Sitt, Jacobo D. Sitt, Harald H. Sitte, Tove Sjögren, Lukasz Skalniak, Mark Skamrahl, **Arne Skerra [4]**, James Slama, Nikolai Slavov, Stephen Sligar, Julia Sliwa, Joan Slonczewski, Judith C. Sluimer, Guy Smagghe, Fatima Smagulova, Jonathan Smallwood, Adam Smith, Brenda Smith, **Deanna Smith [4]**, Kathleen K. Smith, Leslie Smith, Mark Smith, Susan M.E. Smith, Cristian R. Smulski, Lynne Sneddon, Emilie Snell-Rood, William J. Snell, Evan Snitkin, Lawrence H. Snyder, Hon-Cheong So, Shanthini Sockanathan, Olga S. Sokolova, Rusland Soldatov, Rodolfo Solis-Vivanco, Andrey G Solovyev, Mojtaba Soltanlou, Luba Sominsky

Abhijeet R. Sonawane, Charlotte Soneson, Chuliang Song, Gyun Jee Song, Haiwei Song, Hojun Song, Hyun-Seob Song, Ji-Joon Song, Jiuzhou Song, Sen Song, Kai C. Sonntag, Kintake Sonoike, Pierre Sonveaux, **Alice Soragni [4]**, Leonardo Sorci, Mette Sørensen, Carolina Soriano, Philippe Soriano, Natalia Soshnikova, Javier Sotillo, Claudio Soto, Manuel Soto, Laura Soucek, Tewfik Soulimane, Mikle South, Andrew Southam, Marta Souza, Alexandra Sowa, Berna Sozen, András Spaan, Ingrid Span, Konstantin Sparrer, Denis Sparta, Paul Spearman, Doug Speed, Thomas Speltz, Andrew Spence, Jason R. Spence, Nick J. Spencer, Thomas E. Spencer, Robert A. Spicer, Andrew Spiers, Toby Spiribille, Cassandra Spracklen, Thomas C. Sprague, David C. Spray, Simon Sprecher, **Wolfdieter Springer [4]**, John L. Spudich, Jemeen Sreedharan, Miduturu Srinivas, Soumya Srivastava, Jared Stabach, Daniel Stadlbauer, Willem Staels, Jochen Staiger, Jason E. Stajich, Emmanuel A. Stamatakis, Stefan Stanciu, Susan Standring, Matthias Stangl, Danielle Stanisic, Claire E. Stanley, **Robin E. Stanley [6]**, Phillip J. Stansfeld, Brian Stansfield, Ekaterina Stansfield, Jessica Stapley, Lesley Stark, Jeff Staub, Oskar Staufer, Erec Stebbins, Landon Steeg, Christopher Steele, Robert Steele, Gilda Stefanelli, Barbara Stefanska, Ulrike Steffen, Steve Stegen, Brian Steidinger, Matthias Steiger, Gary S. Stein, Lincoln Stein, Reuven Stein, Eike Steinmann, Stefan Stender, Karin Stenkula, Abish Stephen, Amberley D. Stephens, Jessica F. Stephenson, David Stepp, Claudio D. Stern, Jared Sterneckert, Angela Stevenson, Tinna Stevnsner, Alex Stewart, Jason A Stewart, Mark Stewart, Rodney Stewart, Maria Stockenreiter

Johannes Stöckl, Jonathan Stokes, Johannes Stökl, Michael H.B. Stowell, Benjamin Straube, Samantha Streicher, Sergei Strelkov, James Strother, John Strouboulis, Christos Strydis, Natalie Strynadka, Bryan Stuart, Garret Stuber, David J. Studholme, Alexey Stukalov, James Sturgis, Roger Sturmey, Chih-Chia Su, Jin Su, Xin-Zhuan Su, Xuantao Su, Elavarasan Subramani, Lakxmi Subramanian, Carmen C Sucharov, Kumarasamy Sudhakar, Makoto Suematsu, David Suggett, Hiroshi Sugimoto, Sergei S. Sukharev, Petr Sulc, Tabb Sullivan, Pankaj Suman, Seirian Sumner, Dandan Sun, Kang Sun, Ruping Sun, Shi-Yong Sun, Shuying Sun, Wen Sun, Zhenjun Sun, Yoshiaki Sunami, Margaret Sunde, Subramanian Sundharraman, Mark Sundrud, Jaeyun Sung, Jung-Joon Sung, Oriol Sunyer, Fran Supek, Kanya Suphapeetiporn, Dmitry Suplatov, Martha Susiarjo, Mark A. Sussman, Daniel Suter, Gregory Sutton, Mark Sutton, Troy Sutton, Atsushi Suzuki, Ayako Suzuki, Haruo Suzuki, Madoka Suzuki, Masatoshi Suzuki, Norio Suzuki, Shigekatsu Suzuki, Shinsuke Suzuki, Tadashi Suzuki, Toshitaka N. Suzuki, Yasuhiro Suzuki, Yoshiyuki Suzuki, Yutaka Suzuki, Anita Sveen, Mattias N. Svensson, Mikael Svensson, Danielle Swaney, Kendall Swanson, Raymond Swanson, Fredrik J. Swartling, Trevor Sweeney, Russell Swerdlow, Pawel Swietach, Szymon Swiezewski, Matt T. Swulius, Lykke Sylow, Csaba Szabo, Piroska Szabó, Gabor Szalai, Or Szekely, Lóránt Székvölgyi, Miklos Szoboszlay, Rubina Tabassum, Phillippa C. Taberlay, Francisco Taberner, Alessandro Tagliabue, Maurizio Taglialatela, Tomohiko Taguchi, Robert Tague, Shirin Taheri, Stephen Tait, Shinji Takada, Motoki Takaku, Minoru Takata

Takanori Takebe, **Hiromasa Takemura [4]**, Fumihiko Takeuchi, Ratree Takhampunya, Toru Takimoto, Idan Tal, Vincent Tam, Francesco Tamagnini, Marco Tamietto, Gintautas Tamulaitis, Hiroshi Tamura, Choo Hock Tan, Howe-Siang Tan, Hsih Yin Tan, Huiling Tan, Tsutomu Tanaka, Ritesh Tandon, Bo Tang, Chun Tang, Dean G. Tang, Michael Tang, Wanggang Tang, Masateru Taniguchi, John J. Tanner, Weiyang Tao, Maxime Tarabichi, Andrei Tarasov, Jaclyn Taroni, Kaspars Tärs, Sabine Taschner-Mandl, Matthew E. Taylor, Nicholas Taylor, Susan S. Taylor, William Taylor, Peyton Tebon, Wee Wei Tee, Nicholas Teets, Brandon Tefft, Miguel Teixeira, Miguel C. Teixeira, Paulo J. Teixeira, Raphael D. Teixeira, Jesus Tejero, Fabrice Teletchea, Ilknur Telkes, Ryan E. Temel, Daniel Temko, Olivier Tenaillon, Ayse Tenger-Trolander, Anders Tengholm, Maria Tenje, Daniel Tennant, Michaela TerAvest, Akira Terui, M Terunuma, Ryoichi Teruyama, Thomas C. Terwilliger, Jan Tesarik, Ingrid Tessmer, Nehal Thakor, Archana Thakur, Dietmar R. Thal, Roman Thaler, Sebastian Thallmair, Matthias Thalmann, Morten Thaysen-Andersen, Thomas Theil, Francois‐Xavier Theillet, Kevin Theis, Maria Themeli, Jamie Theobald, Clotilde Théry, Michel Thiebaut de Schotten, Jan Christoph Thiele, Meinolf Thiemann, Christoph Thiemermann, Victor Thijssen, Cecile Thirant, Benjamin Thiria, Bolaji Thomas, Charles Thomas, François Thomas, Louise Thomas, Lowri Thomas, Ranjeny Thomas, Andrew Thompson, Christopher Thompson, Damien Thompson, Jeffrey J. Thompson, Linda Thompson, Nathan E. Thompson, Mads S. Thomsen, Aunyaratana Thontiravong, Doreen Thor, Kasper Thorup, Thomas Thumberger, Greg M Thurber, Jingwei Tian, Renmao Tian, Katharine Tibbetts

Denise Tieman, Erik Tihelka, Youri Timsit, David T. Ting, See-Yeun Ting, Jessica Tingle, Philip Tinnefeld, Carine Tisne, Bjoern Titz, Trevor Tivey, Kenneth K.W. To, Fernando Tobias, John A. Todd, George K. Tofaris, Daniel Toker, Gaku Tokuda, Kenzo Tokunaga, Jessica Tollkuhn, Marcelo Tolmasky, Dóra Tombácz, Francesco Tombola, Rafal Tomecki, Jakub Tomek, Shin-ichi Tomizawa, Joseph L. Tomkins, Francesco Tommasino, Shozo Tomonaga, Alessandro Tondelli, Erdal Toprak, Parizad Torabi-Parizi, Giusy Tornillo, Peter Törok, Davis Y. Torrejon, Nicole Torres Tamayo, Pedro Torres-Ayuso, Inma Torres-Campos, Julian Torres-Dowdall, Eduardo M. Torres, Victor Torres, Luis Antonio Tortajada-Genaro, Eneda Toska, Ash Toye, Philip Trackman, Kay Trafford, Patricia Tran, Yara Traub-Csekö, Kyle Travaglini, Gilles Travé, Nicole Trefault, Elizabeth Trembath-Reichert, Marie-Eve Tremblay, Todd Triplett, Madhukar H. Trivedi, Alexandra Trkola, Ras Trokovic, Maurizio Trovato, Megan Troxell, Marta Trub, Sven Truckenbrodt, Jaroslav Truksa, Andrew Truman, George A. Truskey, Feng Chiao Tsai, **Isheng J. Tsai [4]**, Ming-Feng Tsai, Aristidis Tsatsakis, Yu-Hua Tseng, Konstantinos Tsetsos, Lam C. Tsoi, Sophia Tsoka, Constantine D. Tsoukas, Sachio Tsuchida, Hideaki Tsuge, Tomomi Tsunematsu, Hsiung-Lin Tu, Valter Tucci, Raffaele Tucciarelli, Lorena Tuchscherr, Kerry L Tucker, Taru Tukiainen, Damien C. Tully, Sevin Turcan, Ted K. Turesky, Christopher Turner, Julie W. Turner, Silvia Turroni, Lance Turtle, Geetu Tuteja, Lillian Tuttle, Timo Tuuri, Samantha Twiname, Susan Twine, Jeff Twiss, Sonika Tyagi, Silvia Tyciakova, Anna Tyler, William (Jamie) Tyler, Isabel Ubeda-Bañon, Silvia Uccella, Shizuka Uchida

Md Nasir Uddin, Shintaro Uehara, Masaki Ueno, Bret Ulery, Viktor Ulicsni, Peter Ulvskov, Govindhaswamy Umapathy, Hendrik Ungefroren, Atul Upadhyay, Iztok Urbančič, Vlada Urlacher, Karen Usdin, Blake Ushijima, Toshikazu Ushijima, Suayib Üstün, Vladimir N. Uversky, Philip V’kovski, Gustav Vaaje-Kolstad, Alessio Vagnoni, Michael Vahey, Naveen Vaidya, Alexander Vainstein, Patricia Valente, Martina Valentini, Camila Valenzuela, Taufik A. Valiante, Sofie Valk, Sofie L. Valk, Léo Valon, Sandrine Valsesia-Wittmann, Carlos Valverde, Renee Van Amerongen, Jim Van Belzen, Charlene Van Buiten, Eve Van Cauter, Renaud Van Damme, Sebastian Van de Linde, Rieneke Van de Ven-van Balken, Mark F Van Delft, Emile Van Den Akker, Jelle Van den Ameele, Cathelijne W. Van den Berg, Irene Van Den Berg, Adriaan Van der Graaf, Marcel Van der Heijden, Marcel Van der Heyden, Bert Van der Reijden, Emma Van der Westhuizen, Wim Van Drongelen, Nick Van Gastel, Jo Van Ginderachter, Marc Van Goethem, Roel Van Harten, Bennett Van Houten, Laurence Van Melderen, Wynand Van Naters, Johan Van Nes, William E Van Nostrand, Christiaan Van Ooij, Jacobien Van Peer, Wouter Van Rheenen, Ildiko Van Rhijn, Job Van Riet, Bruno Van Swinderen, David L. Van Tassel, Richard Van Wezel, Sivapriya Kailasan Vanaja, Robert VanBuren, Pieter Vanden Berghe, Jamie I. Vandenberg, Barbara Vanderhyden, Jennifer Vandooren, Jennifer Vannest, Daryll Vanover, Rufin VanRullen, Inka Vanwonterghem, Luca Varani, Vitya Vardanyan, Javier Vargas, Rebecca Varney, Ajith Vasanthakumar, Layla Vasquez, Gerardo R. Vasta, Tommi Vatanen, José Vázquez-Prado, Andres Vazquez-Torres, Alexei Vazquez, Veena Veena, Andrea Vega, Silvia Velasco, A Catalina Velez-Ortega, Brandon Velie, Libor Velisek, Jana Veliskova, Lourdes Velo-Suarez, Dan Veltri, Ravichandra Vemuri, Laurent Venance, Miguel Vences, Marc Vendrell

Harry Vennema, Natascia Ventura, Nuria Verdaguer, Victor Vergara, Alexis Verger, Anurag Verma, Paula Vertino, Mihalis Verykokakis, Miguel Vicente-Manzanares, Miguel Vicente, Marcello Vichi, Felipe Viela, Dorothee Viemann, Matam Vijay-Kumar, Nagarjun Vijay, Sukumar Vijayaraghavan, Surendra Vikram, Bjarni Vilhjálmsson, Bjarni J. Vilhjálmsson, Johannes J. Viljoen, Juan Carlos Villarreal, Theresa Vincent, Mark Viney, Kutti R. Vinothkumar, Anne-Clémence Vion, David M. Virshup, Karen Visick, Bryce Vissel, Renee Visser, Flavia Vitale, Alyssa Vito, Aiti Vizzini, Elke Vlemincx, Jakob Voelkl, Annie Vogel Cierna, Beat R. Vogeli, Rufin Vogels, Katrin Vogt, Benjamin F. Voight, Sinisa Volarevic, Waldemar Vollmer, Wolfger Von der Behrens, Eric Von Elert, Astrid Von Mentzer, Annemarie Voorberg-van der Wel, Gary Vora, Alexey Vorobev, Igor Vorobyov, Martin Voskuil, Patrick Vourch, David Vredevoogd, An Vu, Vladyslav V. Vyazovskiy, Jody Vykoukal, Andreas Wack, Eric J. Wagner, Gunter P. Wagner, Nicole Wagner, Ryan Wagner, Fred Wong Wai Shiu, Derek A. Wainwright, Peter Wainwright, Torsten Waldminghaus, Rosie Walker, Kenneth N Wallace, Valerie Wallace, Avner Wallach, Helen Waller-Evans, Kyle M. Walsh, David Walt, Nils G. Walter, Eike-Christian Wamhoff, Edwin Wan, Jiandi Wan, Lin Wan, Zhengpeng Wan, Michal Wandel, Bo Wang, Chengshu Wang, Chunjian Wang, **Daifeng Wang [4]**, Feng Wang, Fengping Wang, Gang Greg Wang, Guangzhou Wang, Guey-Shin Wang, Guocan Wang, Hai-Hong Wang, Hao Wang, Hong Wang, Jia Wang, Jia-Wei Wang, Jincheng Wang, Juexin Wang, Julia Wang, Jun-Tao Wang, Kai Wang, Lei Wang, Liang Wang, Man Wang

Ming-Wei Wang, Minyan Wang, Nan Wang, Peng Wang, Penghua Wang, Ping Wang, Qian-Fei Wang, Rong Wang, Shaomeng Wang, Shaopeng Wang, Shengjun Wang, Shuo Wang, Wen-ming Wang, Wenqin Wang, Xiaoming Wang, Xiaotao Wang, Xifeng Wang, Xinjie Wang, Xu Wang, Yang Wang, Yangming Wang, Yanli Wang, Yejun Wang, Yi Wang, Yin Wang, Yinzhao Wang, Yong Wang, Yu-Chao Wang, Yue Wang, Yujiang Wang, Yuzhuo Wang, Zhao V. Wang, Zhe Wang, Zhen Wang, Zhengfei Wang, Zhenxun Wang, Zhizhi Wang, Phurpa Wangchuk, Christina M Warboys, Maya Wardeh, Juliane Bubeck Wardenburg, Doreen Ware, Ian M. Ware, Shelley Warlow, Philippa Warren, Varun Warrier, Shaun Warrington, Leonhard Waschke, Demian Wassermann, Masashi Watanabe, Rei Watanabe, Takamitsu Watanabe, Takayuki Watanabe, Takeo Watanabe, Yuuki Watanabe, Robert M. Waterhouse, Andrew P. Waters, Rachel Watkins, Andrew J Watson, Corey T. Watson, Jeff Watson, Gary Wayman, Robert Weatheritt, Benjamin Weaver, Lucas Weaver, Jacqueline Webb, Tonya Webb, Richard J. Webby, Kathrin Weber, Brian Weeks, Ann Wehman, Michael Wehr, Chaochun Wei, Guo-Wei Wei, Jin Wei, Lu Wei, Wei Wei, Yao Wei, Yongyi Wei, Thomas Weichhart, Ralph Weidner, Dolf Weijers, Justin Weinbaum, Kevin S. Weiner, Richard Weinkamer, Harel Weinstein, David Weis, Vera Weisbecker, Ellen Weisberg, Steven M. Weisberg, David Weiss, Michael Weiss, Ryan Weiss, Omer Weissbrod, Winfried Weissenhorn, Matthias Weissensteiner, Bernd Weisshaar, Ervin Welker, Haitao Wen, Kenneth Wengler

Esther Veronika Wenzel, Eva Maria Wenzel, Michaela Wenzel, Tamra E Werbowetski-Ogilvie, Danièle Werck-Reichhart, Lars Werdelin, Jan Wessel, Michael Wessels, Alexander J. Westermann, Peter Westh, Urbain Weyemi, Christopher Whalen, Adam K. Wheatley, Matt Wheeler, James White, John H White, Paul Whitford, Jarred Whitlock, Carly Whyte, Max S. Wicha, Amittha Wickrema, Per Widlund, Matthew S Wiebe, Jasmina Wiemann, Wojciech Wiertelak, Stuart Wigby, Ronald Wilders, Craig B. Wilen, Luke Wiley, Doris Wilflingseder, Carol Wilkinson, Holly Wilkinson, Jeremy M. Wilkinson, Karen Willard-Gallo, David C. Williams Jr, Frances M. Williams, Gavin Williams, Jesse Williams, Mark C. Williams, Janna Willoughby, Jonathan Willow, Nicole Wilski, Angus Wilson, Benjamin Wilson, Brenda Wilson, Charles R. Wilson, Emma Wilson, Laurence Wilson, Marcus Wilson, Ross Wilson, Samantha Wilson, Stuart Wilson, Tim Wilson, Danny Winder, Michael Winklhofer, James Winterburn, Wipawee Winuthayanon, Alison Wirshing, Dyann Wirth, Erin Wissink, Janneke Wit, Stephen G. Withers, Adelheid Woehrer, Andrew P. Wojtovich, Damian Wojtowicz, Dana Wolf, Eckhard Wolf, John Wolf, Joanna M. Wolfe, Mariana F. Wolfner, Paweł Wołkow, Isabelle C. Wolowczuk, Knut Woltjen, Tania Wong Fok Lung, Alice Wong, Anderson O.L. Wong, Richard Wong, Yung Hou Wong, Geoffrey Wood, Hollis Woodard, Kristina Woods, Thierry M. Work, **Gordana Wozniak-Knopp [4]**, Christian Wozny, Gregory A. Wray, Naomi R. Wray, Gavin Wright, Antoni Wrobel, Lidia Wrobel, Michael Wrzaczek, Bian Wu, Catherine J. Wu, Chenggang Wu, Haojia Wu, Hongju Wu, Jiang Wu, Jianqiang Wu, Jie Wu, Jinhua Wu, Joae Wu

Liang Wu, Mei X. Wu, Min Wu, Nan Wu, Nicholas Wu, Peng Wu, Wei-Li Wu, Yang Wu, Yiran Wu, Zhengliang L. Wu, Stephan Wuest, Moritz F. Wurm, Claudia Wyer, Saranya Wyles, Doug Wylie, Ursula Wyneken, Tony Wyss-Coray, Nan Miles Xi, Rongwen Xi, Zhiyong Xi, Jiang Xia, Kelin Xia, Tian Xia, Xiao-Jian Xia, Yang Xia, Zhiqiang Xia, Yang Xiang, Ye Xiang, Wan Xiangyuan, Bailong Xiao, Can Xie, Jing Xie, Peng Xie, Ping Xie, Songbo Xie, Zhongjian Xie, Dingliang Xing, Yongna Xing, Wen-Cheng Xiong, Yuguang Xiong, Yuyan Xiong, Wang Xu-Jing, Chi Xu, Haodong Xu, Huaxi Xu, Jin Xu, Mike X. Xu, Nan-Jie Xu, Wenhua Xu, Xiaoji Xu, Yue Xu, Huai-Jun Xue, Xia Xue, Iftach Yacoby, Illya Yakymenko, Kentaro Yamamoto, Masayuki Yamamoto, Yojiro Yamanaka, Hidenori Yamasue, Seiya Yamayoshi, Chaogan Yan, Chongling Yan, Jiayue Yan, Koon-Kiu Yan, Nieng Yan, Riqiang Yan, Shigui Yan, Wei Yan, Bo Yang, Chaoyong Yang, Guang-Yu Yang, Haitao Yang, Hao Yang, Huanming Yang, Jackie (Jiekun) Yang, Jason H. Yang, Jie Yang, Jing Yang, Jinsung Yang, Kai Yang, Lei Yang, Liu Yang, Mu Yang, Qiang Yang, Sheng Tao Yang, Wanling Yang, Wentao Yang, Xiaofeng Yang, Xiaojing Yang, Xue Yang, Xuerui Yang, Zhibo Yang, Ryoichi Yano, Bing Yao, Xuebiao Yao, Hiroyuki Yasuda, Takao Yasui, Michelle Yau, Joseph Yavitt, Deju Ye

Tong Ye, **Kevin Yehl [4]**, Gow-Chin Yen, Ju Hun Yeon, Ali E. Yesilkanal, Joseph Yesselman, Marc Yeste, Guoguo Yi, Peng Yi, Huabing Yin, Xiling Yin, Sheng-Hua Ying, Kwok-Ho Yip, Koji Yonekura, Jun-Ichi Yonemaru, Koichi Yoneyama, Hwan Su Yoon, Sung Ho Yoon, Shige H. Yoshimura, Lingchong You, Alexander Young, Arabella Young, Eric Young, Jared Young, Kendra A. Young, Walter S. Young, Neil A Youngson, Ihab Younis, Han-Gang Yu, Han-Qing Yu, Jianing Yu, Leilei Yu, Manda Yu, Tsyr-Yan Yu, Yuguo Yu, Mengting Yuan, Peng Yuan, Shaochun Yuan, Shuguang Yuan, Wenping Yuan, Zhidong Yuan, Min Yue, Iwata Yuji, Steven Yukl, Hong Wa Yung, Nicholas C Zachos, Roland Zahn, Ran Zalk, Guido Zaman, Maedeh Zamani, Gerald W. Zamponi, Nahuel Zamponi, Hong Zan, Ming-Zhi Zangh, Giuseppe Zanotti, Natalia Zaretskaya, Alon Zaslaver, Mark A. Zaydman, Kristina Zec, Peter Zeidman, Noam Zelcer, Roman Zeleznik, Jian Zeng, Ling-Li Zeng, Ping Zeng, Wenbin Zeng, Karsten Zengler, Weiwei Zhai, Lixing Zhan, Aihui Zhang, Cheng Zhang, Dan Zhang, Di Zhang, Dongshan Zhang, Fan Zhang, Fei Zhang, Haibing Zhang, Haibo Zhang, He-Peng Zhang, Huaye Zhang, Ji Zhang, Jian Zhang, Jianzhen Zhang, Jin Zhang, Jinghui Zhang, Jingyan Zhang, Jisen Zhang, Junjie Zhang, Ke Zhang, Li Zhang, Lianjun Zhang, Lin Zhang, **Lingqiang Zhang [4]**, Linyi Zhang, Meixiang Zhang, Miaomiao Zhang, Ningyan Zhang, Peijun Zhang, Peiyi Zhang, Qinghai Zhang

Qinghua Zhang, Rui Zhang, Sarah Zhang, Shenghong Zhang, Song Zhang, Suyi Zhang, Wenna Zhang, Wenting Zhang, Wenxia Zhang, Xian Zhang, Xiao-Ou Zhang, Xiaoyang Zhang, Xin Zhang, Xing Zhang, Xingtan Zhang, Yiyue Zhang, Yu-Chan Zhang, Yun Zhang, Zeda Zhang, Zhenwen Zhang, Zheying Zhang, Zhihao Zhang, Zhiyan Zhang, Zhongyang Zhang, Allan Zhao, Bo Zhao, Dechun Zhao, Gongpu Zhao, Hien Tran Zhao, Hu Zhao, Huabin Zhao, Huiying Zhao, Jianjun Zhao, Jingjing Zhao, Kai-Hong Zhao, Minglei Zhao, Rui Zhao, Suwen Zhao, Yi Zhao, Zeping Zhao, Shen Zhen, Chunfu Zheng, Jie Zheng, Jing Zheng, Lei Zheng, Siyuan Zheng, Wenjun Zheng, Wenwei Zheng, Yun-Wen Zheng, Zongli Zheng, Jianghong Zhong, Chao Zhou, Haibo Zhou, Hang Zhou, Hengyun Zhou, Jie Zhou, Jin Zhou, Lan Zhou, Lufang Zhou, Ming Zhou, Ming-Ming Zhou, Mowei Zhou, Shengtao Zhou, Wenhua Zhou, Xiang Zhou, Xiao-zhong Zhou, Xin Zhou, Yongjin J. Zhou, Yu Zhou, Yufeng Zhou, Cheng Zhu, Dahai Zhu, Dan Zhu, Fu-Yuan Zhu, Hongtu Zhu, Hua Zhu, Min Zhu, Shu Zhu, Weifei Zhu, Wenhan Zhu, Xiongwei Zhu, Yan Zhu, Zhaozhong Zhu, Ran Zichel, Wilma Ziebuhr, Eduardo Zimmer, Jacques Zimmer, Manuel Zimmer, Russolina Benedeta Zingali, Brian Zingg, Annelies S. Zinkernagel, Martin Zinkernagel, Giovanna Zinzalla, Alessio Zippo, Ahmed Ziyyat, Adam Zlotnick, Michal A. Zmijewski, Janeta Zoldan, Brian D. Zoltowski, Antonio Zorzano, Abba Zubair, Johannes Zuber, Jessica Zucman-Rossi, Inge Zuhorn, Kristen Zuloaga, Xi-Nian Zuo, Robert Zupko, Yves Zurbuchen, Virginia Zurriaguz, Tobias Zust, Arthur Zwaenepoel, Martijn Zwama, Larry S. Zweifel, Jan Zylicz

